# Cellular Heterogeneity During Embryonic Stem Cell Differentiation to Epiblast Stem Cells is Revealed by the ShcD/RaLP Adaptor Protein

**DOI:** 10.1002/stem.1217

**Published:** 2012-09-04

**Authors:** Margherita Y Turco, Laura Furia, Anja Dietze, Luis Fernandez Diaz, Simona Ronzoni, Anna Sciullo, Antonio Simeone, Daniel Constam, Mario Faretta, Luisa Lanfrancone

**Affiliations:** a Department of Experimental Oncology, European Institute of OncologyMilan, Italy; b Department of Life Sciences and Technologies, École Polytechnique Fédérale de Lausanne (EPFL)SV ISREC, Lausanne, Switzerland; c FIRC Institute of Molecular Oncology FoundationMilan, Italy; d CEINGE Biotecnologie AvanzateNaples, Italy; e SEMM European School of Molecular MedicineNaples, Italy; f Institute of Genetics and Biophysics “A. Buzzati-Traverso,” CNRNaples, Italy

**Keywords:** ShcD/RaLP, Embryonic stem cells, Epiblast stem cells, Differentiation, Oct4, Cdx2

## Abstract

The Shc family of adaptor proteins are crucial mediators of a plethora of receptors such as the tyrosine kinase receptors, cytokine receptors, and integrins that drive signaling pathways governing proliferation, differentiation, and migration. Here, we report the role of the newly identified family member, ShcD/RaLP, whose expression in vitro and in vivo suggests a function in embryonic stem cell (ESC) to epiblast stem cells (EpiSCs) transition. The transition from the naïve (ESC) to the primed (EpiSC) pluripotent state is the initial important step for ESCs to commit to differentiation and the mechanisms underlying this process are still largely unknown. Using a novel approach to simultaneously assess pluripotency, apoptosis, and proliferation by multiparameter flow cytometry, we show that ESC to EpiSC transition is a process involving a tight coordination between the modulation of the Oct4 expression, cell cycle progression, and cell death. We also describe, by high-content immunofluorescence analysis and time-lapse microscopy, the emergence of cells expressing caudal-related homeobox 2 (Cdx2) transcription factor during ESC to EpiSC transition. The use of the ShcD knockout ESCs allowed the unmasking of this process as they presented deregulated Oct4 modulation and an enrichment in Oct4-negative Cdx2-positive cells with increased MAPK/extracellular-regulated kinases 1/2 activation, within the differentiating population. Collectively, our data reveal ShcD as an important modulator in the switch of key pathway(s) involved in determining EpiSC identity. Stem Cells*2012;30:2423–2436*

## INTRODUCTION

The Shc (*S*rc *h*omolog and *c*ollagen homolog) family of adaptor proteins is a crucial linker of a plethora of receptors to their downstream effectors [1]. To date, four Shc proteins were identified in mammals and they share a conserved, modular organization with two independent protein binding domains: an amino-terminal phospho-tyrosine binding domain and a carboxyl-terminal Src homology two domain linked by a central glycine and proline-rich region [2]. Despite their high homology in structure, they are implicated in distinct signaling pathways, for example, ShcA p52 and p46 isoforms are implicated in mitogenic and motogenic signaling through the Ras activation promoting cell proliferation and differentiation [3, 4], p66ShcA mediates p53 dependent apoptotic responses through its production of radical oxygen species and the regulation of mitochondrial permeability [5, 6], and ShcC regulates neuronal differentiation and survival through the phosphoinositol-3-kinase signaling [7, 8]. The recently identified fourth member, ShcD/RaLP, has been reported to be expressed in the neuromuscular junction, where it mediates muscle-specific kinase signaling [9], in melanocytes, where it is implicated in mitogen-activated protein kinase (MAPK) signaling [10], and in the brain [9]. ShcD has also been reported to be involved in the migration and invasion of melanoma cells, contributing to the metastatic progression of the disease [10]. However, the physiological function of ShcD is still largely unknown.

Embryonic stem cells (ESCs) are derived from preimplantation embryos of the morula and blastocyst stage [11–13] and they possess two remarkable properties: self-renewal and pluripotency, that is, their ability to differentiate into all cell types deriving from the germ layers, in vivo and in vitro [14–16]. A core transcription network made up of the POU-class 5 transcription factor Oct4, Nanog, and Sox2 cooperatively maintains the circuitry that governs their pluripotency and self-renewal while suppressing the expression of developmental regulators [17–19]. The balance between the levels of these factors is a requirement for the pluripotent state, as their deregulation results in a collapse of the self-renewal network and consequent differentiation of the cells. This is epitomized by Oct4, whose precise levels are fundamental for governing the fate of ESCs: low levels of Oct4 induce trophectoderm differentiation, whereas its overexpression results in the differentiation toward endoderm and mesoderm lineages [19, 20].

ESCs are an invaluable tool to dissect the role of signaling molecules involved in development, as many studies have shown that when they differentiate, the events that occur in vitro faithfully recapitulate those in vivo [21–24]. In order to gain insight into the physiological function of ShcD and based on its high expression in the adult and developing brain [9, 25], we differentiated ESCs to neural lineages and found that ShcD is transiently upregulated during the early time-window of differentiation corresponding to the ESC to epiblast stem cells (EpiSCs) transition and is re-expressed when cells have acquired neural identity.

ESCs have been described as the naïve pluripotent state due to their functional similarity to the preimplantation epiblast [26], whereas EpiSCs represent a distinct state of pluripotency from ESCs. Their pluripotent state has been described as “primed” based on their transcriptional and epigenetic profiles that are most similar to their source, the postimplantation epiblast [26–28]. In fact, EpiSCs express the core pluripotent factors, Oct4, Nanog, and Sox2, but also express early lineage markers such as fibroblast growth factor 5 (Fgf5) and Brachyury, as they are poised for differentiation [27, 28]. The transition from the naïve to the primed state is the initial important step for the commitment to differentiation and the underlying mechanisms are just beginning to be unraveled [29].

It is possible to direct the conversion from naïve to primed pluripotency in vitro under the stimulation of fibroblast growth factor 2 (Fgf2) and Activin and we used this model to investigate the role of ShcD during this transition [30]. Using a novel approach to simultaneously assess pluripotency, apoptosis, and proliferation by multiparameter flow cytometry, we characterized the stages of the acquisition of the primed pluripotent state. We show that ESC to EpiSC transition is a process involving massive cell death and is characterized by a tight coordination between the modulation of Oct4 expression, cell cycle progression, and cell survival. The knockout of ShcD in ESCs resulted in a transient impairment of this differentiation process, revealing the presence of a distinct Oct4-negative caudal-related homeobox 2 (Cdx2)-positive population within the heterogenous composition of ESCs undergoing EpiSC differentiation. Our data show that the absence of ShcD perturbs the commitment process and suggests the implication of ShcD in the switch of key pathway(s) determining EpiSC identity thus providing important insight into the processes occurring during ESC to EpiSC differentiation.

## MATERIALS AND METHODS

### ESC Derivation and Culture

ShcD+/− and −/− ESCs were derived from embryos from ShcD+/− and −/− crossings. Blastocysts were transferred individually to 48 wells with feeder layer of mitotically inactivated mouse embryonic fibroblasts and were cultured in ESC medium: Dulbecco's modified Eagle's medium (DMEM), 15% ESC screened fetal bovine serum (FBS) (PAN), 0.1 mM nonessential amino acids (Life Technologies, Paisley, UK, http://www.invitrogen.com), 2 mM l-glutamine (Life Technologies, Paisley, UK, http://www.invitrogen.com), 25 mM HEPES, 0.1 mM β-mercaptoethanol (Gibco, Life Technologies, Paisley, UK, http://www.invitrogen.com), and leukemia inhibitory factor (LIF; prepared by the Transgenic facility, IFOM-IEO campus) supplemented with 50 μM MAPK/extracellular signal-regulated kinase (MEK1) inhibitor PD98059 (Cell Signaling, Danvers, MA, http://www.cellsignal.com) [31, 32]. Blastocyst outgrowths were passaged in trypsin-EDTA containing 1% chicken serum (Life Technologies, Paisley, UK, http://www.invitrogen.com) onto progressively larger areas with feeder. Established wild-type ESCs E14Tg2a.4 (BayGenomics, http://baygenomics.ucf.edu), Sox1-GFP 46C ESCs [33], and Oct4-GFP ESCs [34] (from Austin Smith, University of Cambridge) and derived ShcD+/− and −/− ESCs were cultured routinely on gelatin under feeder-free conditions in ESC medium or in 2i/LIF medium [35]: N2B27 medium consisting of DMEM/F-12 + Glutamax (Life Technologies, Paisley, UK, http://www.invitrogen.com) and Neurobasal medium without l-glutamine (Life Technologies, Paisley, UK, http://www.invitrogen.com) at a 1:1 mixture, N-2 (Life Technologies, Paisley, UK, http://www.invitrogen.com), B-27 (Life Technologies, Paisley, UK, http://www.invitrogen.com), 0.1 mM β-mercaptoethanol, 2 mM l-glutamine, 25 mM HEPES supplemented with 1 μM MEK inhibitor PD0325901 (Selleck, Houston, TX, http://www.selleckchem.com), 3 μM GSK3 inhibitor CHIR99021 (Selleck, Houston, TX, http://www.selleckchem.com), and LIF. For all experiments, ESCs were used from passage 10 to a maximum of passage 25. Alkaline phosphatase was detected following the manufacture's instructions (Chemicon, Millipore, Billerica, MA, http://www.millipore.com).

### ESC Differentiation

ESCs were differentiated to neural stem cells following the monolayer differentiation protocol [36, 37]. ESCs were seeded onto gelatinized culture dishes at low density (0.5–1.5 × 10^4^ cells per square centimeter) in N2B27 medium. After 8 days, cells were detached in accutase and resuspended in neural stem (NS) expansion medium: Euromed (Euroclone, Milan, Italy, http://www.seuroclonegroup.it) supplemented with 2 mM l-glutamine, N-2, 20 ng/ml of Fgf2 (Peprotech, Rocky Hill, NJ, http://www.peprotech.com), and 20 ng/ml Epidermal growth factor (Peprotech, Rocky Hill, NJ, http://www.peprotech.com), in T25 flasks (Iwaki, Sterilin, Newport, UK, http://www.sterilin.co.uk) for the selection and expansion of neural stem cells. ESCs and differentiating cultures were collected at days 0, 2, 3, 4, 8 and NS cell (NSC) state for expression analysis. EpiSCs were derived from ESCs following the protocol established by Guo et al. [30]. ESCs were seeded onto 15 μg/ml fibronectin (Roche, Milan, Italy, http://www.roche.it) coated six-well plates at 1 × 10^5^ cells per well in ESC medium or in 2i/LIF. After 24 hours, the medium was switched to EpiSC medium: N2B27 supplemented with 12 ng/ml Fgf2 (Peprotech, Rocky Hill, NJ, http://www.peprotech.com) and 20 ng/ml Activin A (R&D systems, Abingdon, UK, http://www.rndsystem.com). When cells reached 80%–90% confluence, they were passaged at high density (typically at 5 × 10^5^ per six-well). ESCs and differentiating cultures were collected at passages 2, 3, 4, 5, and 6 for analysis.

### Multiparameter Fluorescence-Activated Cell Sorting Analysis

Cells were incubated with 10 μm Ethynyl-2′deoxyuridine (EdU) for a pulse of 30 minutes. Cells and their supernatant were collected, fixed in 1% formaldehyde, permeabilized in Triton X-100, and blocked in 1% bovine serum albumine (BSA) in phosphate buffered saline (PBS). Cells were stained for Oct4 (Santa Cruz Biotechnology; sc5279, Santa Cruz, CA, http://www.scbt.com) and cleaved Caspase-3 Asp175 (Cell Signaling; #9661) for 1 hour at RT. Primary antibodies were detected by secondary antibodies goat FITC anti-rabbit (Life Technologies, Paisley, UK, http://www.invitrogen.com) and donkey A647 anti-mouse (JacksonImmunoResearch Europe, Newmarket, UK, http://www.jireurope.com). Cells were processed for EdU detection using the Click-iT EdU Pacific Blue Flow Cytometry Assay Kit (Life Technologies, Paisley, UK, http://www.invitrogen.com) following manufacturer's instructions. Cells were resuspended in PBS containing 2.5 μg/ml propidium iodide (PI; Sigma-Aldrich, St. Louis, MO, http://www.sigmaaldrich.com) and 250 μg/ml RNase (Roche, Milan, Italy, http://www.roche.it) and left at 4°C overnight before acquisition. Samples were acquired using the BD FACS Canto II equipped with laser 405 nm, 488 nm, and 633 nm. Data were analyzed by the BD FACS Diva software version 6.1.1. Cell aggregates were excluded from analysis by appropriate gating of PI fluorescence. All experiments were performed at least in triplicate.

### Immunofluorescence Staining and Confocal Microscopy Analysis

Cells grown on coverslips were fixed in 4% paraformaldehyde (PFA), permeabilized for 10 minutes in 0.1% Triton X-100, and blocked in 2% BSA in PBS. Cells were incubated with either Oct4 (Santa Cruz Biotechnology; sc5279, Santa Cruz, CA, http://wwwscbt.com), Oct3/4-Alexa Fluor 647 conjugated (BD Pharmingen; #560329, Becton Dickinson Italia, Buccinasco, Milan, Italy), Cdx2 (Biogenex; CDX2-88, Fremont, CA, http://www.biogenex.com), or phospho-extracellular-regulated kinases 1/2 (Erk1/2) Thr202/Tyr204 (Cell Signaling, Danvers, MA, http://www.cellsignal.com; #9106 and #4377) for 1 hour at RT. Primary antibodies were detected by secondary antibodies goat FITC anti-rabbit, goat Cy5 anti-mouse (Life Technologies, Paisley, UK, http://www.invitrogen.com), and Alexa Fluor 488 donkey anti-mouse (Jackson). Nuclei were counterstained with 1 μg/ml 4′,6-diamidino-2-phenylindole (Sigma-Aldrich, St. Louis, MO, http://www.sigmaaldrich.com) in PBS. Images were acquired using the Leica SP5 Confocal Microscope at a ×40 1.25 NA oil immersion objective. To preserve statistical significant sensitivity and resolution, large fields of view (>1.2 mm) were acquired with a mosaic approach using the Matrix Routine of the LAS control software (Leica Microsystems, Milano, Italy, http://www.leica-microsystems.com). All experiments were performed at least in duplicate. For further details on experimental procedures, please refer to the supporting information Materials and Methods.

## RESULTS

### *ShcD* Is Expressed Early During ESC Differentiation and Embryonic Development

**Figure 1:**
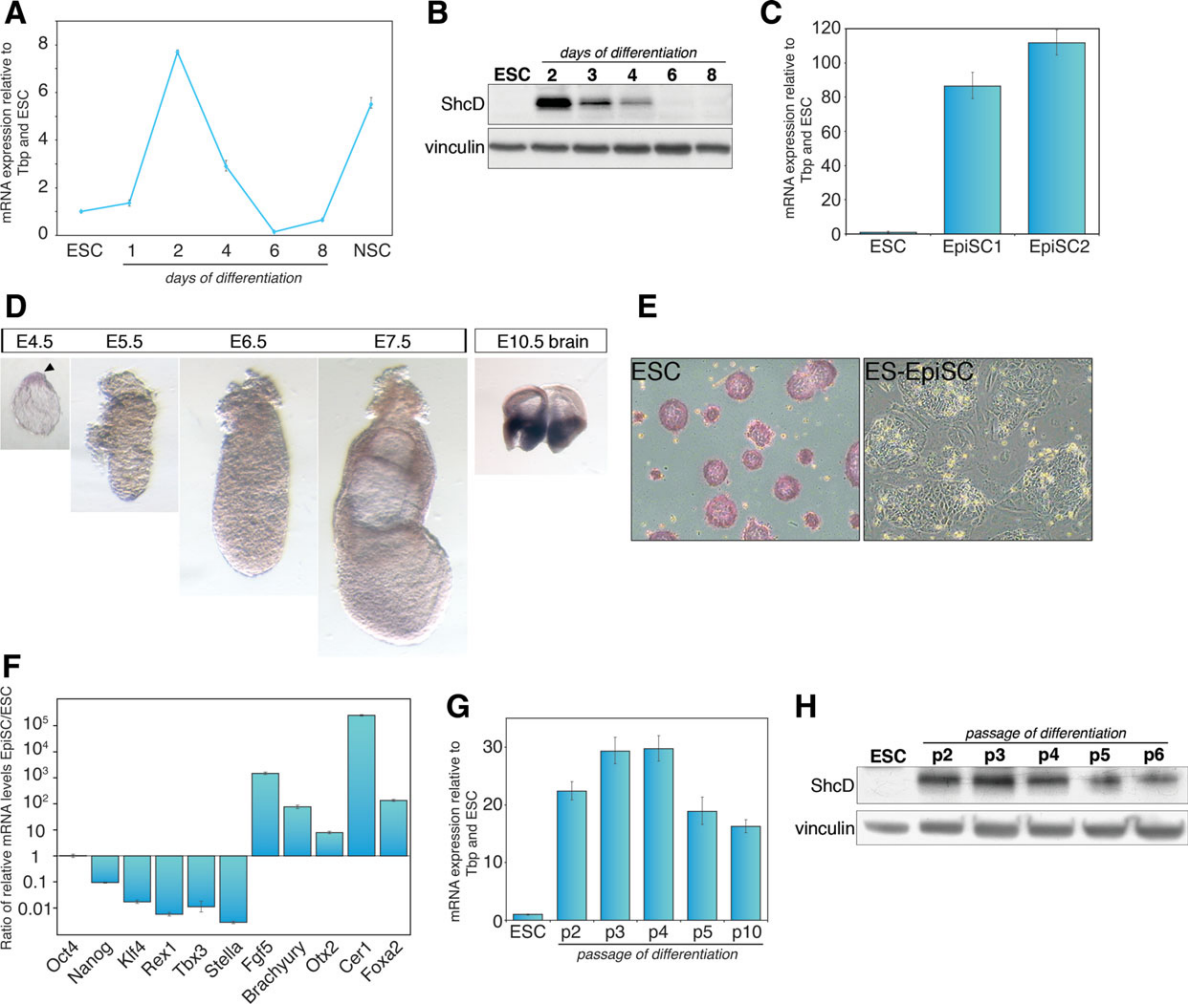
Analysis of *ShcD* expression during ESC differentiation and early embryonic development. **(A):***ShcD* expression analysis by qRT-PCR during neural differentiation of ESCs: ESCs, days 1, 2, 4, 6, and 8 of differentiation and NSCs. ESCs carrying the green fluorescent protein (GFP) reporter under the promoter of the neuroectodermal marker Sox1 (46C) were used to monitor the efficiency of differentiation. *ShcD* expression level in samples was normalized to the *Tbp* and ESCs. Data are represented as mean with ± SD. **(B):** Western blotting for ShcD during neural differentiation of 46C ESCs: ESCs, days 2, 3, 4, 6, and 8 of differentiation. Vinculin is shown as the loading control. **(C):** qRT-PCR for *ShcD* in ESCs, EpiSC lines EpiSC1 and EpiSC2 derived from Oct4-GFP mice. mRNA levels of *ShcD* were determined by normalizing to *Tbp* and ESCs derived from the same mice. Data are represented as mean with ± SD. **(D):** Whole mount in situ hybridization of *ShcD* in early postimplantation embryos at embryonic day E4.5 (peri-implantation), E5.5, E6.5, and E7.5. Whole mount brain preparation of mouse embryo at E10.5 was used as the positive control. **(E):** Bright-field images of cells for alkaline phosphatase staining of cells before (ESC) and at passage 5 of differentiation (ES-EpiSC). Magnification at ×20. **(F):** Gene expression profile of EpiSCs at passage 5 of differentiation when EpiSC cultures are established. The relative expression of each gene is compared to its expression level before differentiation. Bars represent the means ± SD of triplicates. **(G):** qRT-PCR for *ShcD* during ESC to EpiSC differentiation of wild-type ESCs at different passages: ESC, passages 2, 3, 4, 5, and 10. mRNA levels of *ShcD* were determined by normalizing to *Tbp* and ESC. Data are represented as mean with ± SD. **(H):** Western blotting for ShcD during wild-type ESC differentiation into EpiSCs at progressive passages: from ESCs up to passage p6. Abbreviations: EpiSC1, epiblast stem cell 1; ESC, embryonic stem cell; NSC, neural stem cell; *Tbp*, *TATA binding protein*.

It has been previously shown that *ShcD* is expressed at high levels in the adult mouse central nervous system and at low levels in the skeletal muscle [9]. Recently it has also been revealed that it is broadly expressed in the developing nervous system during embryogenesis (supporting information Fig. S1 and [25]). Therefore, we decided to investigate the role of ShcD during the neural differentiation of ESCs under conditions that faithfully recapitulate neurogenic events that occur in vivo [23, 36]. The characterization of *ShcD* expression by qRT-PCR during the neural differentiation of ESCs evidenced a biphasal expression pattern: a transient upregulation within a narrow time-window at the onset of differentiation (day 2) and the de novo upregulation in established NSC culture (Fig. 1A). This transient expression pattern of expression at day 2 of neural differentiation was confirmed at the protein level (Fig. 1B). This was consistent among several ESC lines (data not shown). We were intrigued by the tight control of ShcD expression at the early-stage of differentiation and we decided to focus on understanding its function during this time-window.

When ESCs exit self-renewal and commit to differentiation, they first pass through the primitive ectoderm/EpiSCs stage prior to acquiring the identity of more differentiated lineages [23, 38, 39]. This data are further supported by studies in which EpiSCs, indistinguishable from the postimplantation embryo-derived counterparts, were successfully established from cells during this phase of differentiation [22, 24]. To determine whether *ShcD* expression during this time-window is correlated to EpiSC identity, we performed qRT-PCR on two independently established postimplantation embryo-derived EpiSC lines and found that *ShcD* was indeed highly expressed (Fig. 1C). To investigate the in vivo significance of this modulation, we performed whole mount in situ hybridization for *ShcD* on embryos from the peri-implantation stage at embryonic day (E) 4.5–7.5. We found that *ShcD* expression was detected in the epiblast of the peri-implantation embryo at E4.5 and this expression was transient, as it was no longer detected between E5.5 and E7.5 (Fig. 1D). Taken together, these results suggest a possible physiological function of ShcD during this narrow developmental window when the naïve preimplantation epiblast differentiates into the primed postimplantation epiblast.

The protocol established by Guo et al. directs the differentiation of ESCs into EpiSCs [30], providing an in vitro model to study this transient process. Indeed, EpiSCs derived from ESCs following this protocol were no longer positive for alkaline phosphatase activity (Fig. 1E) and their expression pattern analyzed by qRT-PCR showed the downregulation of naïve epiblast markers such as *Rex1* and *Stella* and the upregulation of early lineage genes such as *Fgf5*, *Otx2*, and *Brachyury* (Fig. 1F), as reported [30]. These EpiSCs could differentiate further into derivatives of the germ layers through the formation of embryoid bodies (data not shown). Importantly, ShcD expression was associated to the acquisition of EpiSC identity, as during the differentiation, it was upregulated with the highest levels during the intermediate passages (p) of the differentiation (p3 and p4) and its expression was maintained once the cells have acquired EpiSC identity by p5 (Fig. 1G). The ShcD protein was also upregulated by p2 and its expression maintained throughout the differentiation (Fig. 1H).

### The Absence of ShcD During ESC to EpiSC Differentiation Results in an Enhanced Apoptotic Selection, Impaired Modulation of Oct4 Expression, and a Decrease in Cell Proliferation

In order to dissect the role of ShcD during the acquisition of EpiSC identity, we established ShcD knockout ESCs using the ShcD knockout mouse model generated by our lab (will be described elsewhere). All ESC lines used for our studies expressed pluripotent stem markers Oct4 and Nanog by immunofluorescence and alkaline phosphatase staining and were karyotypically normal (supporting information Fig. S2A). qRT-PCR analysis of other ESC markers such as *Klf4*, *Rex1*, fibroblast growth factor 4 (*Fgf4*), and *Sox2* were also not affected by ShcD depletion (supporting information Fig. S2B). Finally, we provide evidence that the knockout allele causes complete absence of the ShcD protein (supporting information Fig. S2C). Thorough characterization of established wild-type ESCs under self-renewing conditions (supporting information Fig. S2D, S2E) and during their differentiation to EpiSCs (supporting information Fig. S3) has allowed us to determine that our derived ShcD+/− ESCs behaved in the same manner. As our ShcD+/− ESCs were derived from the same litter as the ShcD−/− ESCs, we show the ShcD+/− lines as our control.

In order to fully characterize the events leading to the generation of EpiSCs, we set up a multicolor flow cytometry protocol for the simultaneous analysis of the pluripotent marker Oct4, cleaved Caspase-3, EdU, and PI. We analyzed the cultures at different passages (p) of the differentiation: ESCs, p2, p3, p4, p5, and p6, to generate snapshots of the culture composition in terms of pluripotent, apoptotic, and proliferating populations, during the acquisition of EpiSC identity.

**Figure 2:**
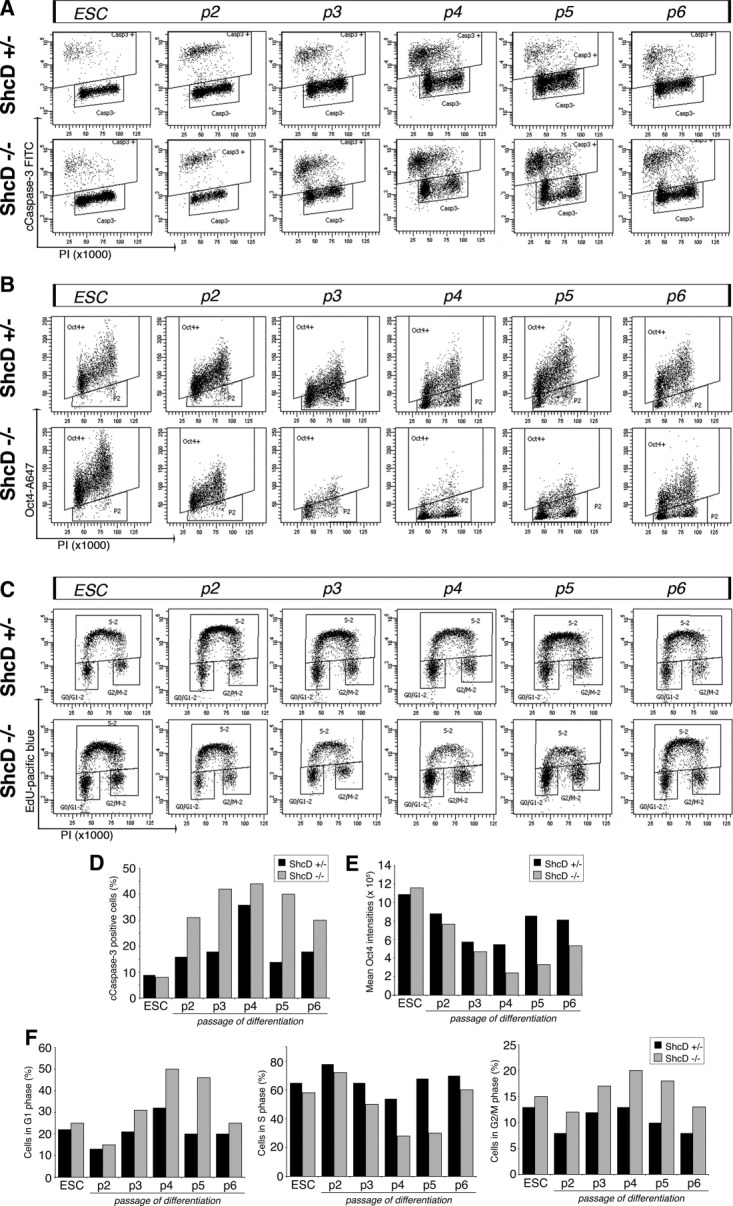
(legend on page 2428).

Analysis of apoptosis by cleaved Caspase-3 and PI staining showed similar low percentages of apoptosis in self-renewing ShcD+/− and −/− ESCs, suggesting no survival impairment from the loss of ShcD in these cells (Fig. 2A, 2D, ESC column). Differentiation toward EpiSC fate was accompanied by a gradual increase of cell death from passage 2 and reaching a peak during the intermediate passages in both ShcD+/− and −/− populations (Fig. 2A). Apoptotic rates then declined once EpiSC colonies were established. The absence of ShcD resulted in a higher apoptotic rate that was prolonged throughout the differentiation, suggesting a reduced cell survival (Fig. 2D).

The cleaved Caspase-3 staining allows not only to assess the apoptotic index but also to analyze the viable population separately from apoptotic cells. This is fundamental for the reliable analysis of other markers as dying cells may have downregulated or lost certain markers. We analyzed the fraction of pluripotent cells at each passage by Oct4 staining, on the viable population (cleaved Caspase-3 negative region; Fig. 2A Casp− box). During the transition of ESCs to EpiSCs, the levels of Oct4 transiently decreased, reaching their lowest mean intensity at intermediate passages and when EpiSC colonies emerged in the late passages, the mean intensity of Oct4 expression returned to levels similar to those in ESC cultures (Fig. 2B, 2E). Although the overall modulation of Oct4 expression in ShcD+/− and −/− cells during the passages of differentiation was similar, the absence of ShcD further enhanced the decline in Oct4 levels during the intermediate passages with a delay in the restoration of the Oct4 expression within the population during the late passages (Fig. 2B, 2E). Thus, the conversion of ESC into EpiSCs involves a tight and coordinated modulation of Oct4 expression, which reaches its minimum in concomitance to the peak of apoptosis, followed by its upregulation after this critical phase (supporting information Fig. S3). Our finding that the removal of ShcD prolongs this period of “crisis” suggests its novel function during ESC to EpiSC transition.

In order to investigate further whether this enhanced crisis observed in the ShcD−/− cultures could be attributed to reduced cell survival or different proliferative capacities, we analyzed the incorporation of the thymidine analog, EdU in the viable population (cleaved Caspase-3 negative) during the differentiation. We observed that during ESC differentiation to EpiSCs, the cell cycle profile of the cells was also modulated (Fig. 2C). The cell cycle distribution of ESCs revealed an enrichment of cells in S-phase with minor contribution from G1 and G2/M fractions, confirming that most of the doubling time was spent for DNA synthesis (Fig. 2C, 2F; supporting information Fig. S2D). Similar percentages were observed in the absence of ShcD, suggesting no dramatic alterations in the proliferative capacity in the ESC state. In contrast, during the intermediate passages of differentiation toward EpiSC state, the fraction of replicating cells decreased with a concomitant increase in the proportion of cells in G1-phase and to a lesser extent in G2/M phase (Fig. 2F). This striking shift in the cell cycle profile was more accentuated in the absence of ShcD, as could be seen by a twofold lower percentage and a lower EdU intensity of cells in S-phase, and a higher fraction of cells in G1 and G2/M phases during the intermediate passages.

Multiparameter flow cytometric analysis thus revealed that, independently from the analyzed genotype (wild-type, ShcD+/− and −/− cells), the differentiation toward the EpiSC state is characterized by multiple concomitant events: (a) massive cell death, (b) temporary arrest or delay in proliferation marked by a transient G1 accumulation, and (c) downregulation of Oct4 expression within the population prior to acquiring EpiSC fate. We found that the peak of these events and the establishment of EpiSC cultures could vary from one to two passages and therefore experimental replicates were not combined. Based on our results, we have subdivided the stages of differentiation process into: early passage (p2), intermediate passages (p3 and p4), and late passages (p5 and p6). These processes occurring during ESC to EpiSC differentiation were clearly unmasked in the ShcD−/− cells as they presented higher apoptosis and prolonged recovery of Oct4 expression for the acquisition of EpiSC state during the intermediate passages.

### Cdx2-Positive Cells Emerge During ESC to EpiSC Differentiation and the Absence of ShcD Results in a Higher Fraction of these Cells Within the Population

**Figure 3:**
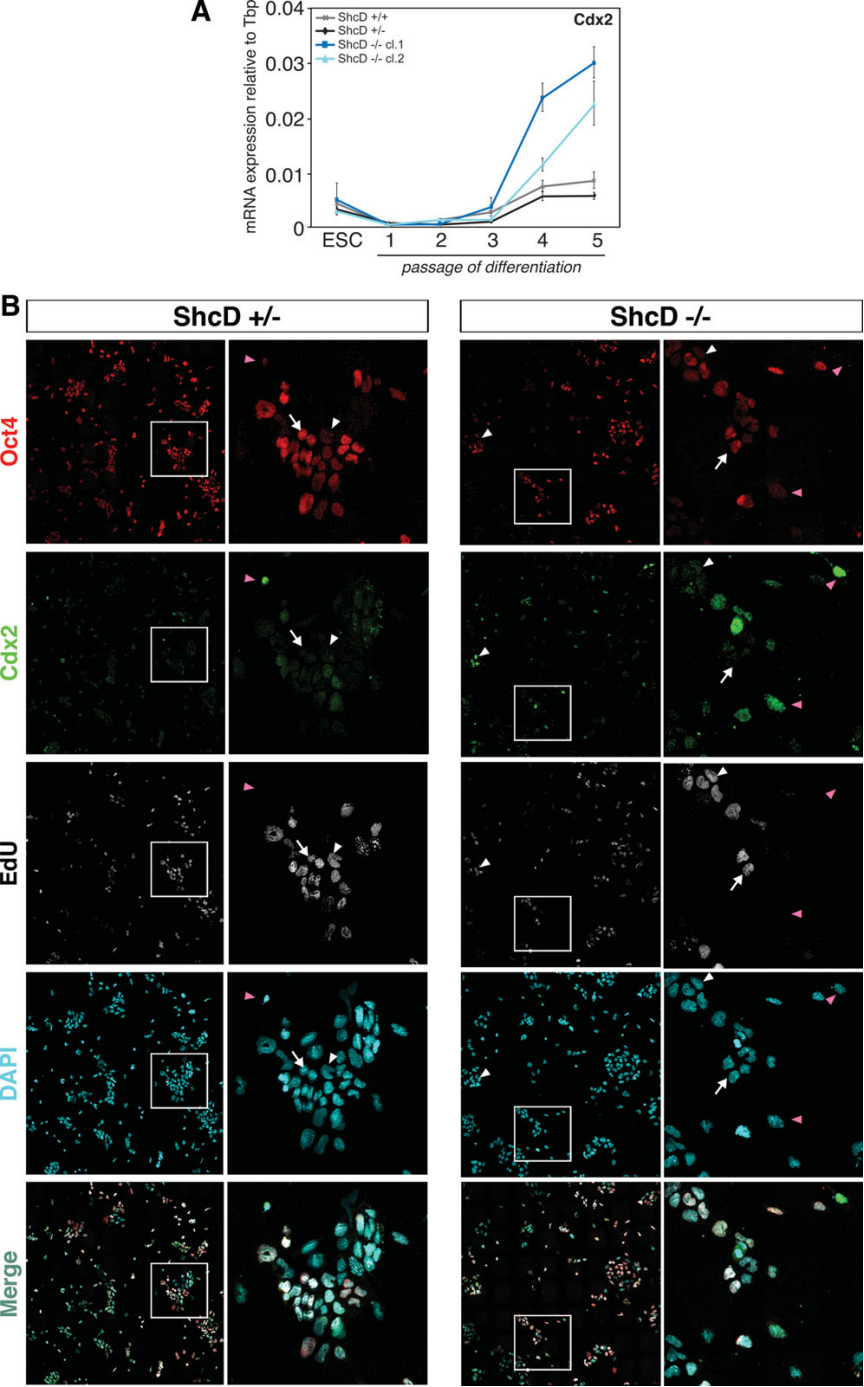
Cdx2-expressing cells emerge during ESC to epiblast stem cell (EpiSC) differentiation and the absence of ShcD results in a higher presence of these cells. **(A):***Cdx2* expression by qRT-PCR on wild-type, ShcD+/− and −/− cells during ESC to EpiSC differentiation. For ShcD−/− cells, two independently derived clones (cl.1 and cl.2) are shown. Expression levels were normalized to the *TATA binding protein* and ESCs. Data are represented as mean with ± SD. **(B):** High-content immunofluorescence analysis for Cdx2, Oct4, EdU, and DAPI on an intermediate passage (p4) of differentiation of ShcD+/− and −/− ESCs. For each genotype, left column shows images taken at ×40 and assembled with mosaic approach. Right column shows zoom-in images of the inset area from the corresponding left image. White arrowheads indicate Oct4-low or negative cells, magenta arrowheads indicate Oct4-negative/Cdx2-positive cells, and arrows indicate Oct4-positive cells. Abbreviations: DAPI, 4′,6-diamino-2-phenylindole; EdU, ethynyl-2′deoxyuridine; ESC, embryonic stem cell.

With the progression of differentiation, an increasing fraction of Oct4-negative cells was revealed, peaking at intermediate passages. When the bulk of the differentiating population then began to restore Oct4 levels, the presence of a fraction of cells that did not became evident revealing the heterogeneity of the population (Fig. 2B, box P2). The emergence of this Oct4-negative population was also detected in the wild-type and ShcD+/− cultures, whereas the ShcD−/− cultures showed a higher presence. To address what might be the nature of this heterogeneity, we analyzed a panel of lineage markers by qRT-PCR of the cultures during differentiation. Interestingly, the *Cdx2* transcription factor that marks the extraembryonic trophectoderm lineage [40] was consistently upregulated during the differentiation in the ShcD−/− cultures (Fig. 3A). Smaller variations in controls confirmed that the phenomenon was not de novo generated by the absence of ShcD but greatly enhanced in response to it. By contrast, the expression of other markers such as those specific of ESCs (*Rex1*, *Klf4, Fgf4*, and *Nanog*), primitive endoderm (*Gata6*), and mesoderm (*Brachyury*) during ESC to EpiSC differentiation showed similar expression patterns in ShcD−/− cells compared to controls (supporting information Fig. S4).

Cdx2 is involved in the trophoblast lineage specification at the blastocyst stage in mice and its expression is intricately linked to Oct4, as they exhibit reciprocal inhibition [40, 41]. Cdx2 represses Oct4 to promote extraembryonic fate, while Oct4 is required to repress this fate. To further investigate the putative correlation between Oct4 and Cdx2 in this context, we performed high-content microscopy analysis to assess Oct4, Cdx2 expression, and EdU incorporation. Mosaic acquisition allows the collection of a wide field of view by the tiling of neighboring images while ensuring optimal single-cell magnification and sensitivity at the highest possible optical resolution [42]. Thus, expression of target genes can be analyzed on a statistically significant number of cells while maintaining morphological information, such as colony formation, which is lost during fluorescence-activated cell sorting (FACS) analysis.

During the differentiation, we observed two distinct cell types based on their Oct4 expression and morphological characteristics: cells that highly express Oct4 growing in clusters typical of EpiSC colonies (Fig. 3B, arrows) and cells that expressed low levels or were negative for Oct4 that at the border of the colonies or growing in isolation (Fig. 3B, arrowheads). A fraction of the Oct4-negative cells expressed Cdx2 and their presence within the ShcD−/− differentiating population increased during the intermediate passages (Fig. 3B, magenta arrowheads). Higher Cdx2 expression in ShcD−/− cultures was also followed by a marked upregulation of other trophoblast markers such as *Elf5*, *Eomes*, and *Gata3* during an intermediate passage of differentiation (supporting information Fig. S5A). Furthermore, *Hand1,* a marker of a more differentiated trophoblast cell type, was also increased in the absence of ShcD (supporting information Fig. S5B).

**Figure 4:**
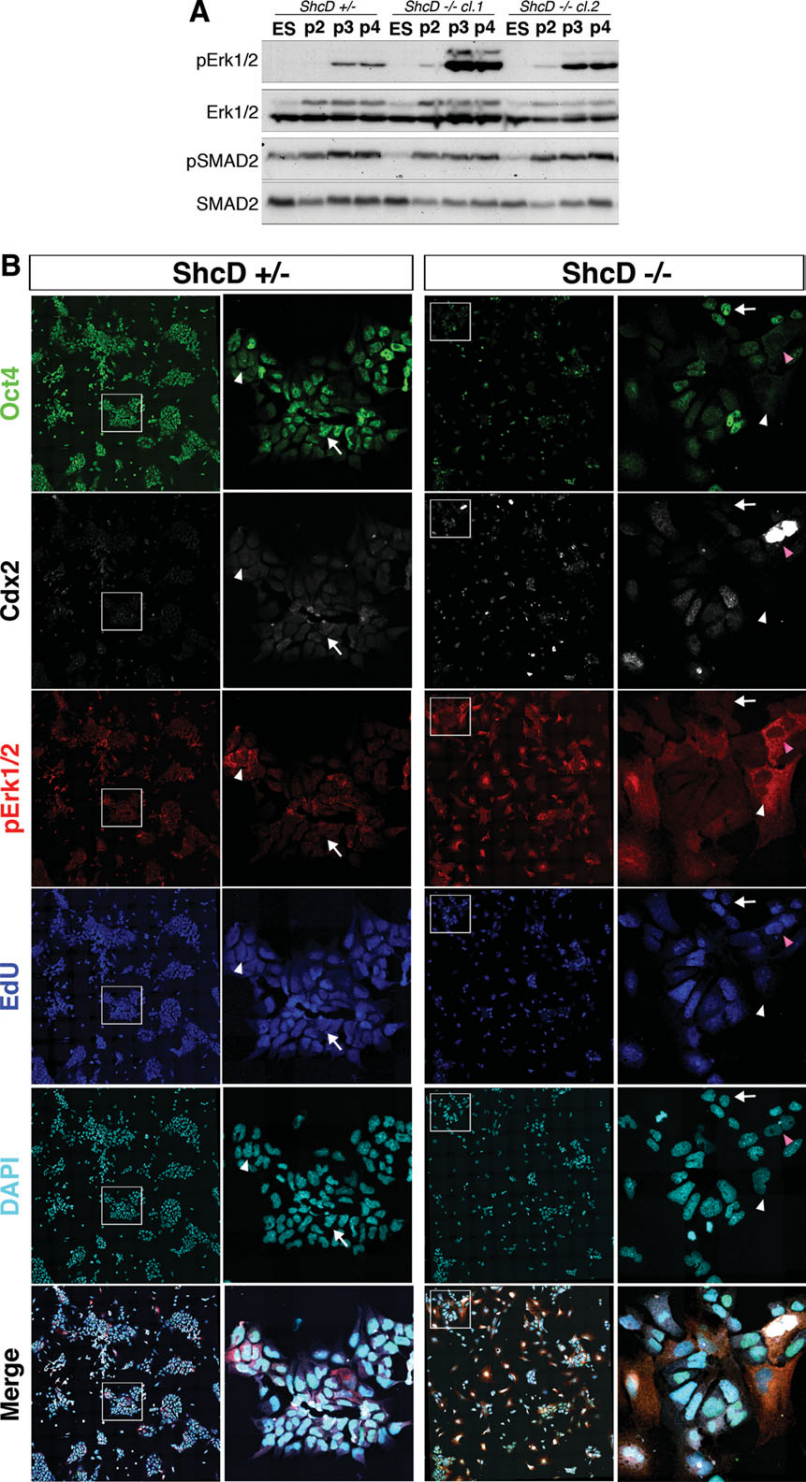
Cdx2-positive cells show high levels of phospho-Erk1/2 and the absence of ShcD results in overall higher levels of phospho-Erk1/2. **(A):** The levels of phospho-Erk1/2 and phospho-SMAD2 during ESC to epiblast stem cell differentiation of ShcD+/− and −/− ESCs were analyzed in starting population of ESCs, passages 2, 3, and 4 of differentiation by Western blotting. **(B):** High-content immunofluorescence analysis for Cdx2, phospho-Erk1/2, EdU, and DAPI on an intermediate passage (p4) of differentiation. For each, left column shows images taken at ×40 and assembled with mosaic approach. Right column shows zoom-in images of the inset area from the corresponding left image. Arrows indicate cells with low levels of pErk1/2 activation that correlates with Oct4-positivity and arrowhead indicates cells with higher levels of pErk1/2, in white for cells that are Oct4-low or negative and in magenta for Oct4-negative/Cdx2-positive cells. Abbreviations: DAPI, 4′,6-diamino-2-phenylindole; EdU, ethynyl-2′deoxyuridine; Erk1/2, extracellular-regulated kinases 1/2; ES, embryonic stem.

During the intermediate passages of differentiation, cells negative for Oct4 showed ongoing DNA replication (Fig. 3B and supporting information Fig. S6). A fraction of cells within the Oct4-negative Cdx2-positive population was also EdU positive, indicating that they do have proliferative ability (Fig. 3B, magenta arrowheads in entire field of view images, and Fig. 4B, magenta arrowheads). In the absence of ShcD, the presence of the Oct4-positive colonies was decreased while the fraction of Cdx2-positive cells was more evident, suggesting that the perturbation of its signaling pathway during the conversion of ESC to EpiSCs results in an enrichment of this cell type, enhancing the heterogeneity of the population (supporting information Fig. S7A).

### Cdx2-Positive Cells Present During ESC to EpiSC Differentiation Have Higher Levels of Phospho-Erk1/2

ESCs and EpiSCs rely on different signaling pathways for their self-renewal in vitro: ESCs depend on the LIF-signal transducer and activator of transcription 3 and bone morphogenetic protein 4 (BMP4) signaling, whereas EpiSCs are dependent on FGF-Erk1/2 and Nodal/Activin A-Smad2/3 signaling [27, 28]. Therefore, the conversion of ESCs to EpiSCs requires the acquisition of specific modulators to rewire the different signaling pathways to the pluripotency circuit. To focus on the mechanisms altered during the differentiation in the absence of the ShcD adaptor protein, we examined the activation status of these two pathways. Western blot analysis of the differentiating cultures showed that the kinetics of phospho-Smad2/3 accumulation paralleled the observed Oct4 regulation, with increasing levels with passaging and were comparable in ShcD+/− and −/− cells (Fig. 4A). However, phospho-Erk1/2 levels increased during the differentiation and were consistently higher in the absence of ShcD, compared to the controls. The overall higher pErk1/2 level in the ShcD−/− culture during an intermediate passage was evident also by immunofluorescence (supporting information Fig. S7B).

Considering the heterogeneous composition of the cell population during the differentiation, phospho-Erk1/2 signaling levels could differ between the cell types. We consequently applied multiparameter microscopy analysis to clarify the scenario. We found that cells of smaller size that expressed high levels of Oct4 and growing in colonies (arrows) had lower levels of phospho-Erk1/2 activation compared to the fraction of cells expressing negative for Oct4 or expressing it at low levels (white arrowheads) (Fig. 4B). Furthermore, within the Oct4-negative fraction, cells that expressed Cdx2 had also high levels of pErk1/2 (Fig. 4B, magenta arrowheads), consistent with reports that *Cdx2* is downstream to MAPK signaling [43]. At late passages when EpiSC cultures were established, the overall pErk1/2 levels were similar between control and ShcD−/− cells and in fact, Cdx2-positive cells also gradually disappeared by this stage of differentiation with the cultures consisting in a majority of Oct4-positive cells (supporting information Fig. S8).

### Cdx2-Expressing Cells Are Present in ESC Cultures

Recent studies demonstrated that ESCs under pluripotency-preserving conditions possess an intrinsic transcriptional signature that gives rise to biological heterogeneity [44]. A subpopulation that is able to originate a fully competent endoderm-like lineage has also been demonstrated [45]. In agreement with this observation, we noticed that the Oct4-negative compartment was present in comparable percentages under cell culture conditions preserving self-renewal and pluripotency of ESCs in both in ShcD+/− and −/− cells (Fig. 2, box P2).

To clarify whether the presence of the Cdx2-positive population represents an innate heterogeneity in the biological program of ESCs, we decided to investigate the origin, behavior, and fate of these cells using an independently derived ESC line. We used a high-content live-cell imaging approach on an in vitro reporter system, an ESC line expressing green fluorescent protein (GFP) under the control of the Oct4 promoter [34]. To temporally correlate the reciprocal relation between Cdx2 and Oct4, we immunostained the cells after live-cell imaging and with the use of a reference grid, traced them retroactively to identify the Cdx2-positive cells and to reconstruct their proliferative history. It should be noted that our image tiling approach screens an active field of view of several millimeter and therefore allows the observation of a large number of cells, including those that are rare within the population.

**Figure 5:**
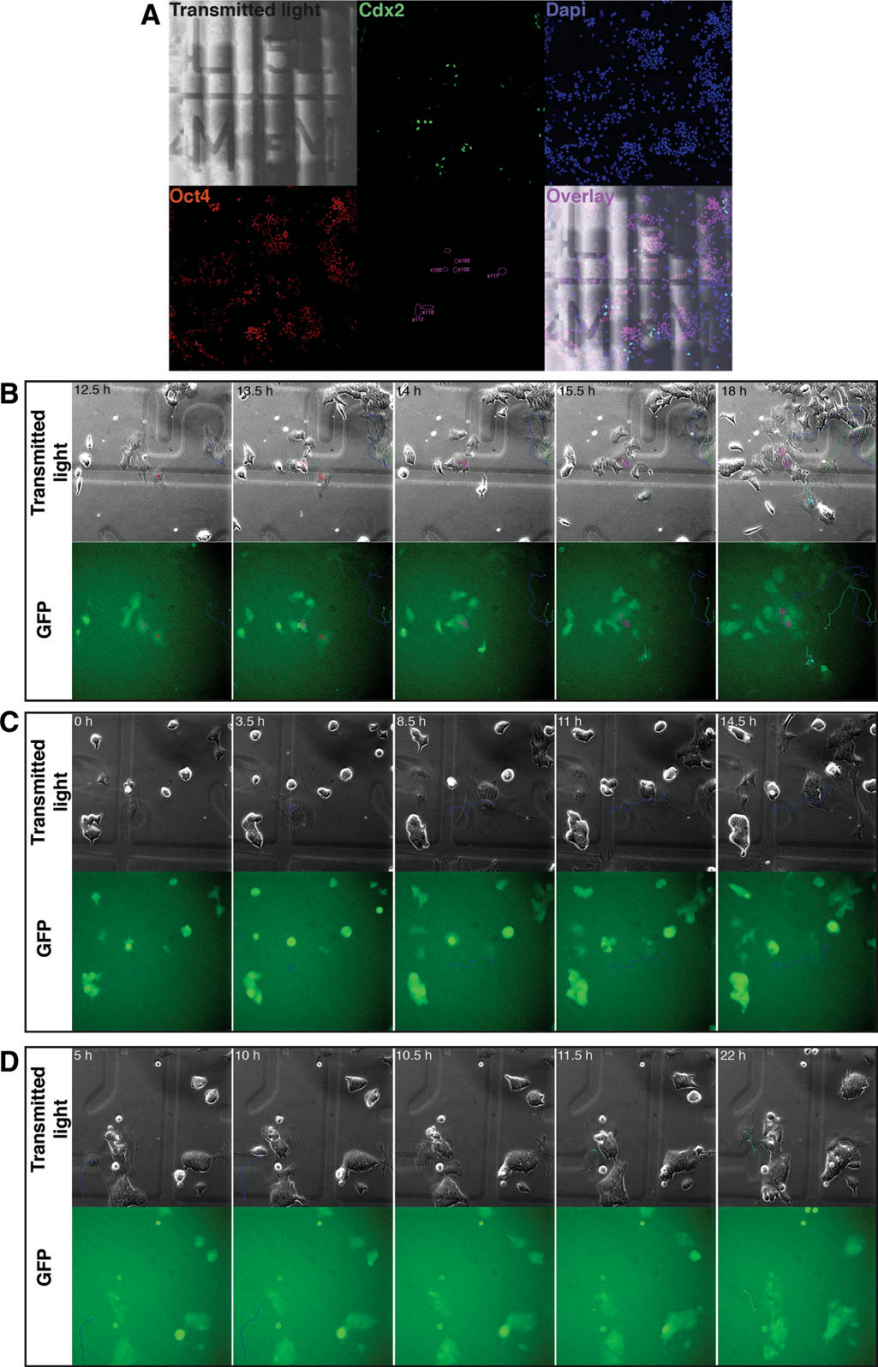
Cdx2-expressing cells are present in embryonic stem cell (ESC) cultures and have distinct migratory and morphological characteristics from Oct4-positive cells. An ESC line expressing GFP under the control of Oct4 promoter cultured under ESC conditions was imaged by wide-field microscopy with ×20 magnification using fluorescence optics. Images were taken every 30 minutes for 24 hours. **(A):** Cells were immunostained after live-cell imaging for Cdx2 and Oct4, using image tiling approach to gain an active field of view of several millimeters and through the presence of a reference grid, reidentified the Cdx2-positive cells to reconstruct their proliferative history. **(B):** Selected images from supporting information Movie 1. Time points indicated in hours. The migratory paths of observed cells are traced with a line and the current position with a dot. Three different behaviors with respect to GFP expression can be observed from this movie: Cells that maintain GFP positivity (marked in pink), cells that are initially GFP-positive and become negative (marked in red then in cyan), and cells negative for GFP that remain negative (marked in blue and green). **(C):** Selected images from supporting information Movie 2. GFP-positive and -negative cells exhibit distinct morphological and migratory characteristics. Cells are growing either as colonies (GFP positive) or sparsely (weak GFP expression or absent) in culture. An example of a GFP-negative cell (marked in blue) that does not maintain contact with other cells, migrates extensively while gradually increasing its size, in contrast to GFP-positive cells that are smaller in size, grow in tightly packed colonies, and show little migration. **(D):** Selected images from supporting information Movie 3. A fraction of GFP-negative cells undergo endoreduplication, giving rise to polyploid cells. The enlarged cell not engaged in colony formation (marked in blue) detaches and reattaches without cell division resulting in a cell containing two nuclei (marked in green). Abbreviations: DAPI, 4′,6-diamino-2-phenylindole; GFP, green fluorescent protein.

First, we confirmed that during normal ESC culture and independently from the strain originating the cell line, a small proportion of cells negative or weakly expressing Oct4 were present and that a fraction of these cells (<1% over the entire population) expressed Cdx2 (Fig. 5A). In accordance to the previous data described even if reduced, there was active DNA replication and thus proliferation of the Oct4-negative cells, as we could monitor several cell divisions even in absence of GFP expression (supporting information Movie 1). Second, when we traced several Oct4-negative cells back to their original source, we found that they arose from cells expressing GFP that progressively lost activity in the Oct4 promoter. This suggests that the Oct4-negative cells are not generated from a clonal expansion of a previously present negative compartment (Fig. 5B and supporting information Movie 2). Examination of the relative spatial localization of the GFP-positive and -negative cells revealed that the distribution of GFP-negative cells coincided with the scattered position of Cdx2-positive cells observed during ESC to EpiSC differentiation. Furthermore, the GFP-positive cells proliferated, maintaining close contact with surrounding cells while in contrast, the GFP-negative cells showed an enhanced migratory activity and did not engage in colony formation (Fig. 5C and supporting information Movie 3). In addition, we observed frequent endoreduplicating events of the GFP-negative cells that led to the generation of tetraploid cells (Fig. 5D and supporting information Movie 4). Although the tetraploid population maintained high migratory ability, they progressively ceased replicating activity and did exhibit Cdx2 positivity, albeit in a fraction of these cells. Thus, our high-resolution live-cell imaging approach enabled the identification of a distinct Cdx2-positive population within ESC cultures that could account for the observed presence and transient expansion of these cells during ESC to EpiSC differentiation.

## DISCUSSION

This study revealed a role for the ShcD/RaLP, a new member of the Shc family of adaptor proteins, during the conversion of ESCs to EpiSCs. This phase of transition from the naïve epiblast to the primed epiblast is very difficult to study in vivo due to limited accessibility and low temporal precision of the epiblast in this short window of time. The protocol recently published in 2009 by Guo et al., which allows the commitment of ESCs to EpiSCs [30], provides a useful tool to dissect the mechanisms involved in this process. Our studies on the ESC to EpiSC differentiation using the ShcD−/− model, combined with a novel approach of multiparameter FACS and high-content immunofluorescence analysis, have allowed us to simultaneously analyze several events during this differentiation in a statistically significant manner, providing insight into a previously uncharacterized process.

Oct4 plays a pivotal role in self-renewal, pluripotency, and lineage commitment of ESCs [19, 46, 47]. Oct4 is also expressed in EpiSCs [27, 28] and interestingly, we found that during the transition of ESCs to EpiSCs in vitro, Oct4 levels are initially downregulated in the differentiating population and then restored when the cells have acquired EpiSC identity. It is possible that the protocol simply selects for EpiSCs already present within the ESC cultures. However, the decrease in Oct4 levels within the entire population during the intermediate passages argues against this interpretation. Furthermore, we observed similar kinetics of Oct4 modulation during the differentiation of ESCs grown in standard ESC medium as well as in 2i/LIF medium, a stringent culture condition that does not allow the survival of other cell types. Therefore, we propose that EpiSC cultures are established through an active process in which ESCs differentiate to acquire this primed pluripotent state. In parallel, cells unable to undergo this conversion and/or differentiate to alternative cell types are eliminated through apoptosis.

During the differentiation of ESCs to EpiSCs, we observed the presence of two populations based on their Oct4 expression: Oct4-positive cells and cells expressing low levels or negative for Oct4. The use of our ShcD−/− model, which showed a transient increase in apoptosis, reduction in proliferative ability, and delay in the restoration of Oct4 levels, evidenced the heterogeneity of the population during the intermediate passages of differentiation. We found that a subset of the Oct4-negative cells expressed Cdx2, a marker for extraembryonic trophectoderm. This finding was unexpected because studies have shown that cells expressing Cdx2 can be generated from ESCs only upon the genetic manipulation of key transcription factors, for example, overexpressing Cdx2 [41], depletion of Oct4 [19], or by deleting Sox2 [48] or the methyltransferase Dnmt1 [49]. In addition to the positivity to Cdx2, their morphological criteria such as their flat morphology, enlarged cell size, clear appearance of cytoplasm, position at the border of the colonies, and high migratory behavior suggest a possible trophoblast lineage of the cells. The presence of endoreduplication events seen by the progressive accumulation of cells in the G2M-4N tetraploid compartment also seems to support this interpretation. There are a few reports that support our view that wild-type ESCs can give rise to trophoblast-like cells from ESC cultures by the specific manipulation of cell culture conditions [50, 51] and Hemberger et al. have also shown the presence of cells expressing the transcript for *Esrrb*, another trophoblast marker in wild-type ESC cultures [52]. Furthermore, a recent study has shown that within the ESC cultures, a rare population of totipotent cells that can contribute to both embryonic and extraembryonic tissues (2C cells) exist [53]. The rare presence of Oct4-negative Cdx2-positive cells within the ESC population that our approach of high-content microscopy allowed us to detect is in agreement with this observation. However, *Cdx2* is not a gene expressed solely in the trophoblast, it is also an early mesodermal marker [54]. In fact, a recent study has shown, although in human ESCs and murine EpiSCs, that trophoblast-associated genes including Cdx2 can be induced upon BMP treatment and these cells share key features with mesoderm (and possibly extraembryonic mesoderm) [55]. Therefore, a careful analysis for various trophoblast markers such as Esrrb and Eomes for trophoblast stem cells, Pl1 and Pl2 for trophoblast giant cells as well as mesodermal markers on the identified Cdx2-positive cells, is necessary to accurately define the lineage of these cells.

Several scenarios can be proposed as the source of Cdx2-expressing cells during the acquisition of EpiSC fate: ESCs, an intermediate population generated during the differentiation, or cells that have acquired EpiSC identity. Although the presence of a fraction of Oct4-negative cells is a feature of EpiSC populations [56, 57], we could not detect Cdx2 positivity within the postimplantation-derived EpiSCs and late passages of ES-derived EpiSC cultures (data not shown). Thus, the last scenario can be excluded. Time-lapse imaging of Oct4-GFP ESCs coupled with immunostaining for Cdx2 revealed that they arise from cells initially expressing Oct4. This suggests that Cdx2 cells are not a result of clonal expansion of a contaminating side population and is in agreement with previous studies that its expression is correlated to Oct4 [41]. Heterogeneity is an intrinsic characteristic of ESCs [17, 44, 45, 58, 59] and our results suggest that the presence of a rare fraction subpopulation expressing Cdx2 is also a characteristic of the ESC cultures.

**Figure 6:**
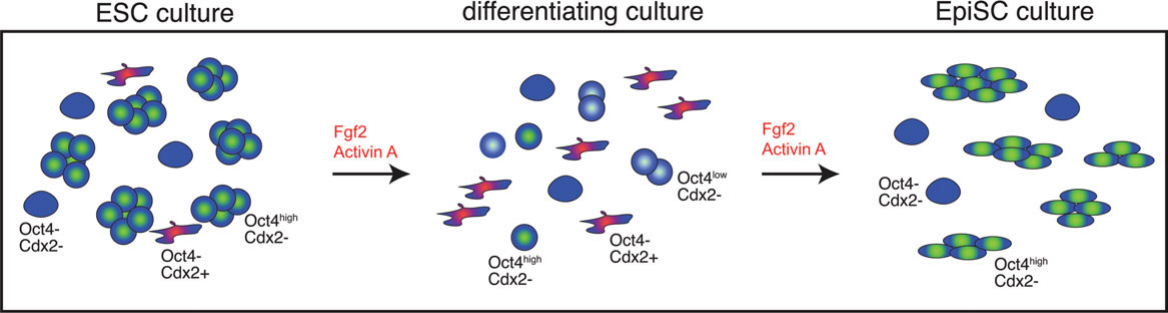
Proposed model of processes occurring during the differentiation of ESCs to EpiSCs. ESC cultures contain a small fraction of Oct4-negative cells and a rare subpopulation of these cells expresses Cdx2. During the differentiation of ESCs to EpiSCs, the differentiating ESCs downregulate Oct4 and undergo a temporary cell cycle arrest. The Oct4-negative Cdx2-positive cells are unaffected by the culture conditions and continue to proliferate leading to a temporary enrichment of these cells within the population. The cells that are not competent to undergo differentiation are eliminated by apoptosis. As the differentiation competent ESCs acquire EpiSC identity, they have reacquired Oct4 expression and proliferative ability and overtake the culture. These processes occurring during ESC to EpiSC differentiation were clearly unmasked in the ShcD−/− cells as they were enhanced and prolonged prior to the recovery and acquisition of EpiSC state. Abbreviations: EpiSC, epiblast stem cell; ESC, embryonic stem cell.

Taken together, our data suggest a model that describes two different phenomena occurring within the cultures during the differentiation of ESCs to EpiSCs (Fig. 6). First, the differentiation conditions cause the temporary downregulation of Oct4 in the differentiating cells, followed by the restoration of its levels as the cells acquire EpiSCs identity. Second, within the Oct4-negative cells generated by ESCs, a subset of cells expressing Cdx2 are transiently enriched during the intermediate passages as they are largely unaffected by the differentiation conditions while the Oct4-positive compartment undergoes temporary cell cycle arrest. This effect was greatly enhanced in the ShcD−/− cultures, unmasking the presence of the Oct4-negative Cdx2-positive cells. While the cells competent to give rise to the EpiSC population reacquire proliferative ability in concomitance with their re-expression of Oct4, the Cdx2-positive compartment seems to undergo a differentiation program generating tetraploid cells and leading to the loss of their proliferation capacity and progressive disappearance from the population through passaging. Thus, the proliferative EpiSCs overtake the culture, resulting in an established EpiSC culture consisting of a majority of Oct4-positive cells. This model does not exclude the presence of other cell types in established EpiSC cultures, as a constant presence of Oct4 negative cells has been reported by others [60].

Cdx2 expression has been shown to be downstream of high Erk1/2 activation [43] and we also found this correlation during the ESC to EpiSC differentiation. Furthermore, the absence of ShcD resulted in an enhanced activation of phospho-Erk1/2 during the differentiation. Previous studies in melanoma cells have shown that ShcD binds to Grb2 to mediate the Ras-MAPK pathway and Grb2 has also been shown to be important for lineage segregation between the epiblast and the primitive endoderm in the embryo [10, 61]. In the context of ESC to EpiSC differentiation, our data support the view that the effect of enhanced phospho-Erk1/2 in the ShcD−/− cultures is indirect. We have observed that Cdx2-expressing cells have higher levels of phospho-Erk1/2 compared to the Oct4-expressing cells and that the ShcD−/− cultures contain a larger proportion of Cdx2-postive cells within the cultures with respect to the controls. Therefore, suggesting that most likely the enhanced levels of pErk1/2 mirror the composition of the culture. Further studies are needed to address this aspect and to collocate ShcD within a signaling pathway in this model system. Recent studies have demonstrated the pivotal role of Wnt/β-catenin signaling during the transition of ESC to EpiSCs [29, 62], this pathway would be certainly one of the candidates for establishing the mechanism of ShcD-mediated signaling.

In summary, our ShcD−/− genetic model was a valuable tool to dissect diverse and complex phenomena occurring during the transition of ESCs to EpiSCs. ShcD absence in ESCs under self-renewing conditions did not cause an effect; however, under differentiation conditions, a transient impairment in the establishment of the EpiSC lineage was revealed. Although the absence of ShcD did not create a de novo phenotype, it resulted in a perturbation of this transition due to higher levels of apoptosis, a dramatic drop in Oct4 expression, and a delayed reconstitution of Oct4 levels within the differentiating population revealing the heterogeneity generated during the differentiation.

## CONCLUSION

Our results, together with the specific expression window of ShcD in the peri-implantation epiblast, suggest that ShcD plays an important role in the switch of a key pathway(s) involved in determining EpiSC identity. Defining the molecular determinants involved in the acquisition of pluripotent states and in the conversion of different stem cell types is fundamental to be able to harness stem cell behavior, we have shown for the first time the involvement of an adaptor protein, ShcD/RaLP, in the acquisition of EpiSC identity.

## Supplementary material

Supplementary Figure 1. Radioactive in situ hybridization for ShcD in E10.5, E12.5 and E16.5 mouse embryos. The upper row shows sagittal sections and the lower row shows transverse sections of the embryos, at different stages. Regions of signal are indicated by abbreviations and/or white arrowhead. In summary, ShcD transcripts were evidenced in the dorsal root ganglia, cranial ganglia and regions of the developing brain. Abbreviations: anterior hypothalamus (ahy), basal ganglia (ba), cephalic ganglion V (V), cephalic ganglion IX (IX), cerebellum (cb), cerebellar precursors (cbp), cortex (cx), primordial cochlea (co), dorsal aorta (da), diencephalon (di), dorsal root ganglia (drg), dorsal thalamus (dt), forebrain (fb), intestines (gu), internal ear (ie), kidney (ki), liver (li), lungs (lu), mandible (ma), midbrain (mb), mesencephalon (ms), olfactory bulb (ob), olfactory epithelium (oe), optic vesicle (ov), pineal gland (pi), pons (po), rostral migratory stream (rms), septum of forebrain (se), telencephalon (te), tongue (to).

Supplementary Figure 2. Characterization of wild-type and derived ShcD+/− and −/− ESCs. (A) Wild-type, ShcD+/− and −/− clone 1 (cl.1) and clone 2 (cl.2) lines that were employed for the studies were characterized for embryonic stem cell (ESCs) markers. Panels from left to right: bright field picture of ESCs grown on inactivated feeder cells; alkaline phosphatase staining; immunofluorescent images of Oct4 and merged with DAPI; immunofluorescent images of Nanog merged with DAPI. Scale bar represents 50 μm. Karyotype analysis show normal karyotype of ESCs as reverted images of DAPI. Red dots indicate chromosomes and the total number of chromosomes (2n=40) is indicated in red at the top left corner of each image. (B) qRT-PCR for pluripotency markers, Rex1, Klf4, Fgf4 and Sox2 on wild-type and derived ShcD+/− and −/− lines, grown under self-renewal conditions in ESC medium. mRNA levels of the markers are shown relative to TATA Binding protein (Tbp) levels for each sample. Bars represent the means ± S.D. of triplicates. (C) Western blotting for ShcD in the ESC lines before and after EpiSC differentiation. Ponceaus S staining is shown as the loading control for the blot. (D) Summary of cell cycle phase distribution of the ESC lines analyzed by FACS with propidium iodide (PI) staining. Cells were grown under self-renewal conditions. Bars represent the means ± S.D. of triplicates. (E) Analysis of apoptosis within the ESC cultures under standard selfrenewal conditions by FACS analysis of the sub-G1 population by PI staining. Bars represent the means ± S.D. of triplicates.

Supplementary Figure 3. Multiparameter FACS analysis of cleaved Caspase-3 expression, Oct4 expression and cell proliferation in wild-type ESCs during their differentiation to EpiSCs. Wild-type ESCs were differentiated to EpiSCs and the cultures were analyzed for cleaved Caspase-3, Oct4, ethynyl-2′deoxyuridine (EdU) and propidium iodide (PI) by FACS, at several stages of the differentiation: ESCs, early passage (p2), intermediate passages (p3 and p4) and late passages (p5 and p6). (A) Dotplots of cleaved Caspase-3 staining (columns two and four are dot plots with FITC intensities on the y-axis and PI on the x-axis) during the differentiation. (B) Cleaved Caspase-3 negative cells were analyzed for Oct4 expression. Shown are the dot plots with A647 intensities on the y-axis and PI on the x-axis. Line divides the mean intensity below which cells are considered to be negative for Oct4. (C) The proliferative ability of wild-type ESCs undergoing EpiSC differentiation was assessed by analyzing EdU incorporation together with PI staining, on the viable population (cleaved Caspase-3 negative) and separately for the Oct4-positive and negative population. Shown are dot plots with Pacific Blue intensities on the y-axis and PI on the x-axis. The top row shows dotplots for Oct4-positive population and the bottom row for Oct4-negative population. Shown here are the results of a representative FACS experiment as the timing of the onset of these described phenomena could vary slightly between experiments.

Supplementary Figure 4. Analysis of pluripotency and lineage markers during ESC to EpiSC differentiation. qRT-PCR profiles of pluripotency and early lineage genes on wild-type, ShcD+/− and −/− ESCs during EpiSC differentiation. mRNA levels of the markers are shown relative to Tbp levels for each sample. Bars represent the means ± S.D. of triplicates.

Supplementary Figure 5. Analysis of extraembryonic markers during ESC to EpiSC differentiation. (A) qRT-PCR for Elf5, Eomes and Gata3 in ShcD+/− and −/− cells at intermediate passage (p4) of differentiation. The relative expression levels for these markers have been normalized to Tbp and bars represent the means ± S.D. of triplicates. (B) qRT-PCR for Hand1 in wild-type, ShcD+/− and −/− cells during ESC to EpiSC differentiation. Its relative expression level was normalized to Tbp and bars represent the means ± S.D. of triplicates.

Supplementary Figure 6. Multiparameter FACS analysis of cell proliferation in Oct4 positive and negative cells by EdU incorporation during ESC to EpiSC differentiation. In order to assess the proliferative capacities of the Oct4-positive and negative cells, the two populations were analyzed separately for their EdU incorporation. Oct4-positive ShcD+/− cells (columns one and two from the left) and Oct4-negative ShcD+/− cells (columns three and four from the left) analyzed for EdU in ESCs, two (p2), three (p3) and four (p4) of differentiation. Columns one and three show the cell cycle of viable cells analyzed (Caspase-3 negative). Columns two and four are dot plots with Pacific Blue intensities on the y-axis and PI on the x-axis. The cell cycle phases were gated according to their EdU positivity and negativity, and DNA content based on their PI intensity. In ESCs, the majority of the cells expressing Oct4 are cycling cells while the majority of the Oct4-negative cells were EdUnegative and in the G0/G1 or G2/M phases. Upon differentiation, both the Oct4- positive and negative populations contain cycling cells. In the ShcD−/− cultures there was a lower proportion of cells that have incoporated EdU in both the Oct4-positive and negative populations.

Supplementary Figure 7. In the absence of ShcD, Cdx2-positive cells and overall activation of pErk1/2 are higher. (A) Single immunostaining for Cdx2 on ShcD+/− and −/− cultures during an intermediate passage (p4) of differentiation. Top row shows the merge of Cdx2 and Dapi images taken at 40X and assembled with mosaic approach. Bottom row shows zoom-in images of the inset area from the corresponding top image. The ShcD−/− population contains a majority of cells growing in isolation and expressing Cdx2 (arrowheads). In the ShcD+/− culture, most cells are growing in tight colonies that are negative for Cdx2. A few cells Cdx2- positive cells are present also in the ShcD+/− culture, occurring at the border of the colonies (arrowhead) or growing in isolation. (B) Single immunostaining for pErk1/2 on ShcD+/− and −/− cultures during an intermediate passage (p4) of differentiation. Top row shows the merge of pErk1/2 and Dapi images taken at 40X and assembled with mosaic approach. Bottom row shows zoom-in images of the inset area from the corresponding top image. Cells occurring at the border of the colonies or growing in isolation express high levels of pErk1/2 (arrowheads). The majority of the cells in the ShcD−/− population showed higher activation of pErk1/2.

Supplementary Figure 8. At a late passage of ESC to EpiSC differentiation, ShcD+/− and −/− cultures have overall similar levels of pErk1/2. High-content immunofluorescence analysis for Oct4, Cdx2, pErk1/2, EdU and DAPI on late passage (p6) of differentiation of ShcD+/− and −/− ESCs. For each genotype, images taken at 40X and assembled with mosaic approach are shown. At p6 Cdx2 cells are scarse also in the −/− cultures and the overall activation levels of pErk1/2 are similar.

Supplementary Movie 1. Time-lapse movie of Oct4-GFP ESCs grown under standard culture conditions imaged by wide-field microscopy with 20X magnification using fluorescence optics. Images were taken every 30 mins for 24 h. The migratory paths of observed cells are traced with a line and the current position with a dot. A cell expressing low levels of GFP (marked in blue) loses GFP expression completely. However, it retains the capacity to proliferate and undergo cell division, giving rise to another GFP-negative cell (marked in green).

Supplementary Movie 2. Time-lapse movie of Oct4-GFP ESCs grown under standard culture conditions imaged by wide-field microscopy with 20X magnification using fluorescence optics. Images were taken every 30 mins for 24 h. The migratory paths of observed cells are traced with a line and the current position with a dot. Three different behaviours with respect to GFP expression can be observed from this movie: Cells that maintain GFP positivity (marked in pink), cells that are initially GFP-positive and become negative (marked in red then in cyan), and cells negative for GFP that remain negative (marked in blue and green).

Supplementary Movie 3. Time-lapse movie of Oct4-GFP ESCs grown under standard culture conditions imaged by wide-field microscopy with 20X magnification using fluorescence optics. Images were taken every 30 mins for 24 h. The migratory paths of observed cells are traced with a line and the current position with a dot. GFP-positive and negative cells exhibit distinct morphological and migratory characteristics. An example of a GFPnegative cell (marked in blue) that does not maintain contact with other cells, migrates extensively while gradually increasing its size, in contrast to GFP-positive cells that are smaller in size, grow in tightly packed colonies and show little migration.

Supplementary Movie 4. Time-lapse movie of Oct4-GFP ESCs grown under standard culture conditions imaged by wide-field microscopy with 20X magnification using fluorescence optics. Images were taken every 30 mins for 24 h. The migratory paths of observed cells are traced with a line and the current position with a dot. A portion of GFP-negative cells undergo endoreduplication giving rise to polyploid cells. The enlarged cell not engaged in colony formation (marked in blue), detaches and reattaches without cell division resulting in a cell containing two nuclei (marked in green).
