# Supplementary figures and images for: Cellular Heterogeneity During Embryonic Stem Cell Differentiation to Epiblast Stem Cells is Revealed by the ShcD/RaLP Adaptor Protein
Source: Stem Cells. 2012 Sep 4;30(11):2423–36. doi: 10.1002/stem.1217 (PMC3533801; doi:10.1002/stem.1217)

E 10.5

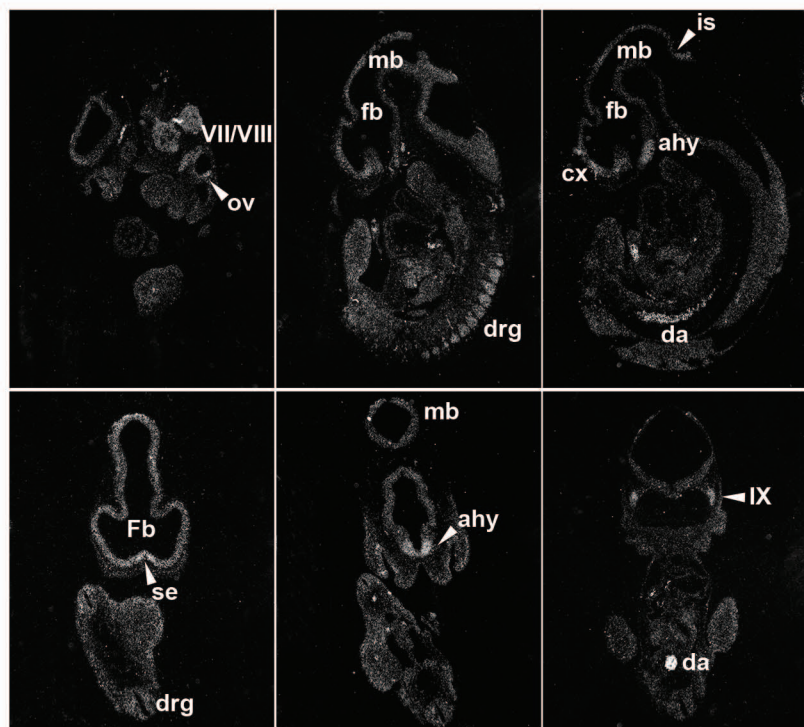

E 12.5

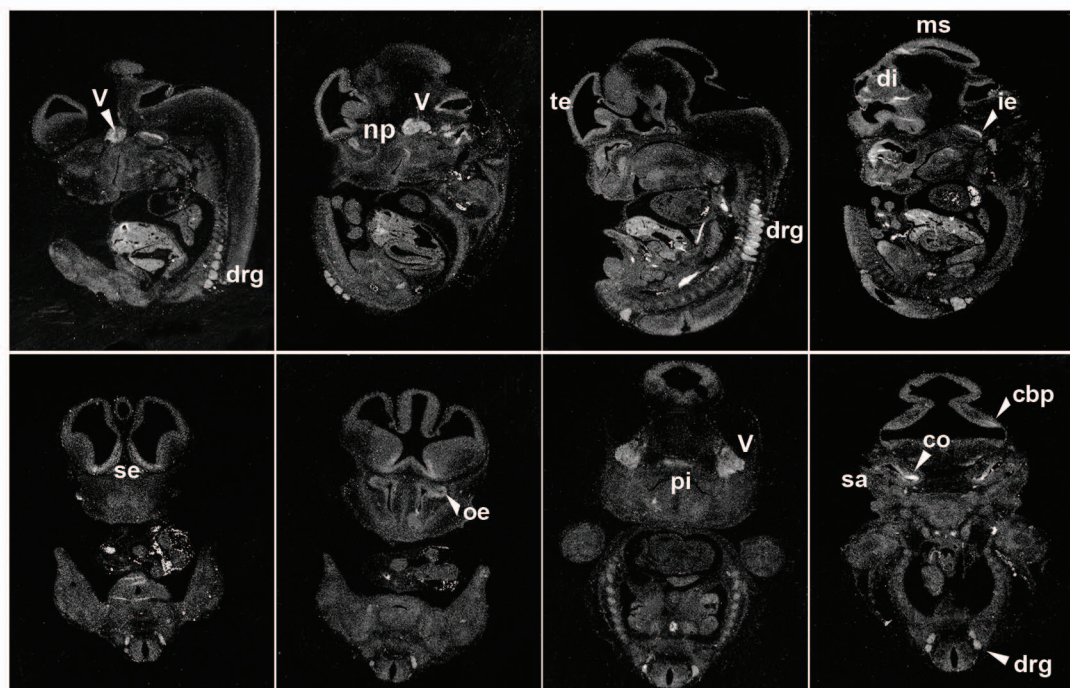

E 16.5

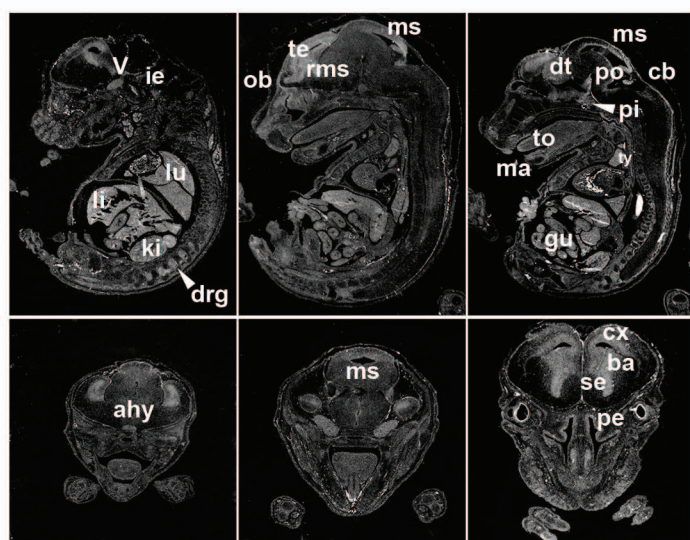

Supplement: Supplementary file 1 [file stem0030-2423-SD1.pdf]

**A**

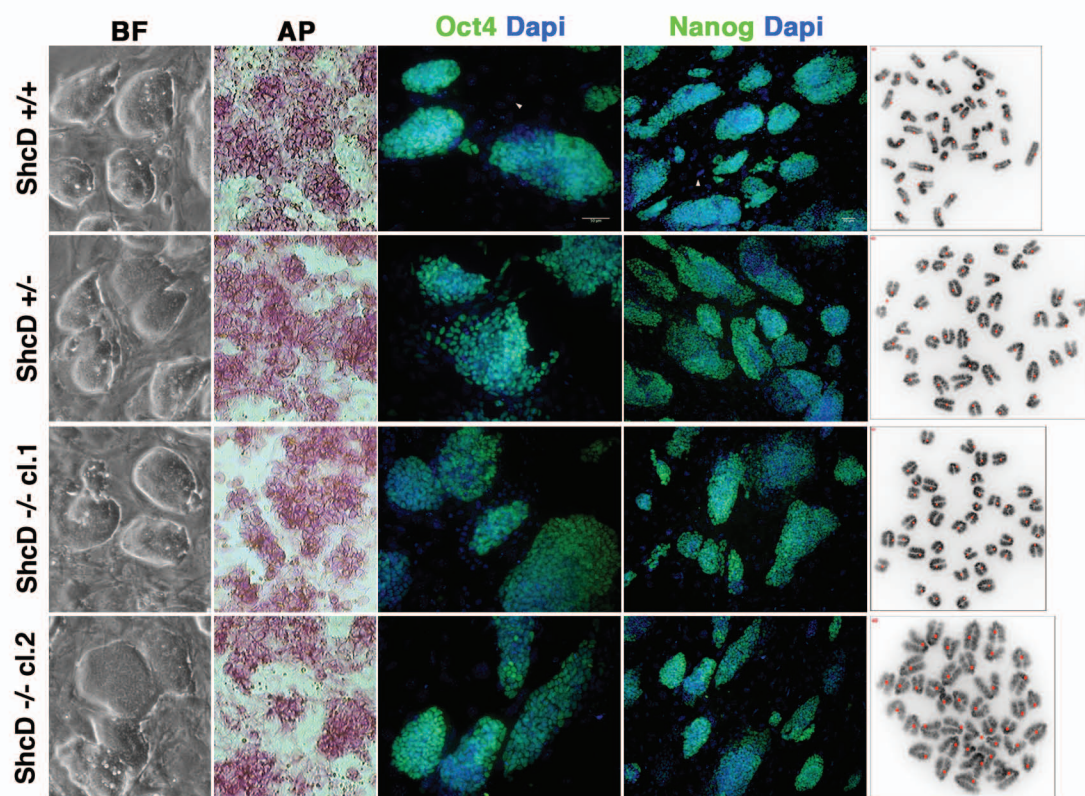

**B**

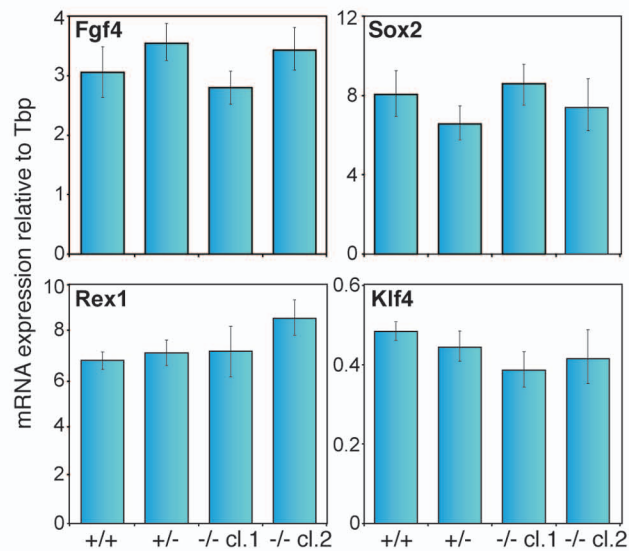

**D**

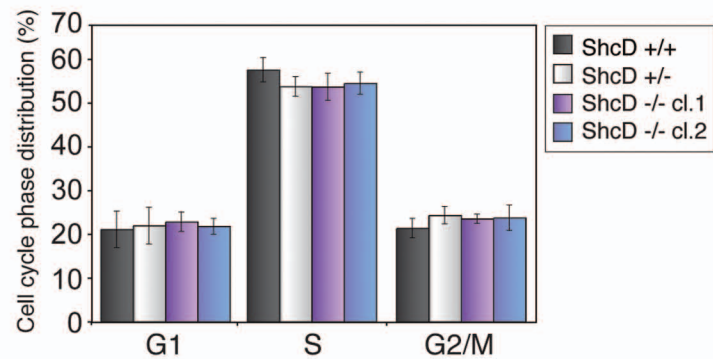

**E**

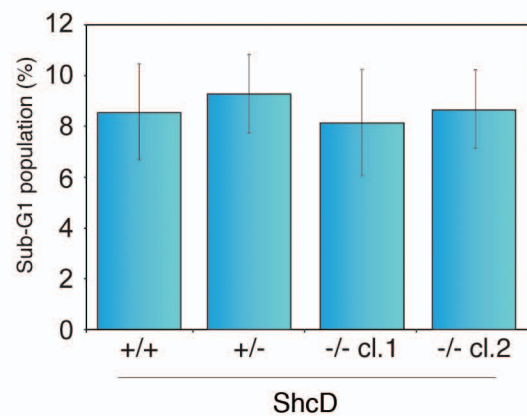

**C**

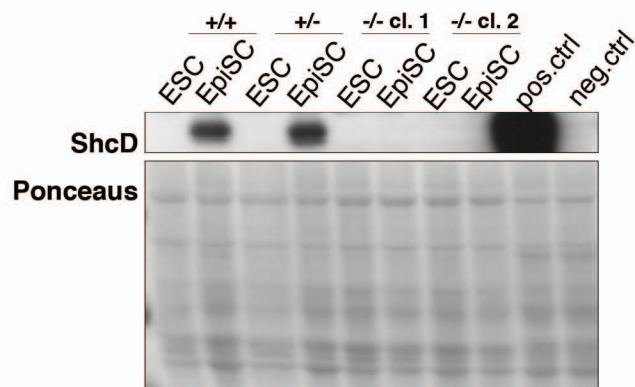

Supplement: Supplementary file 2 [file stem0030-2423-SD2.pdf]

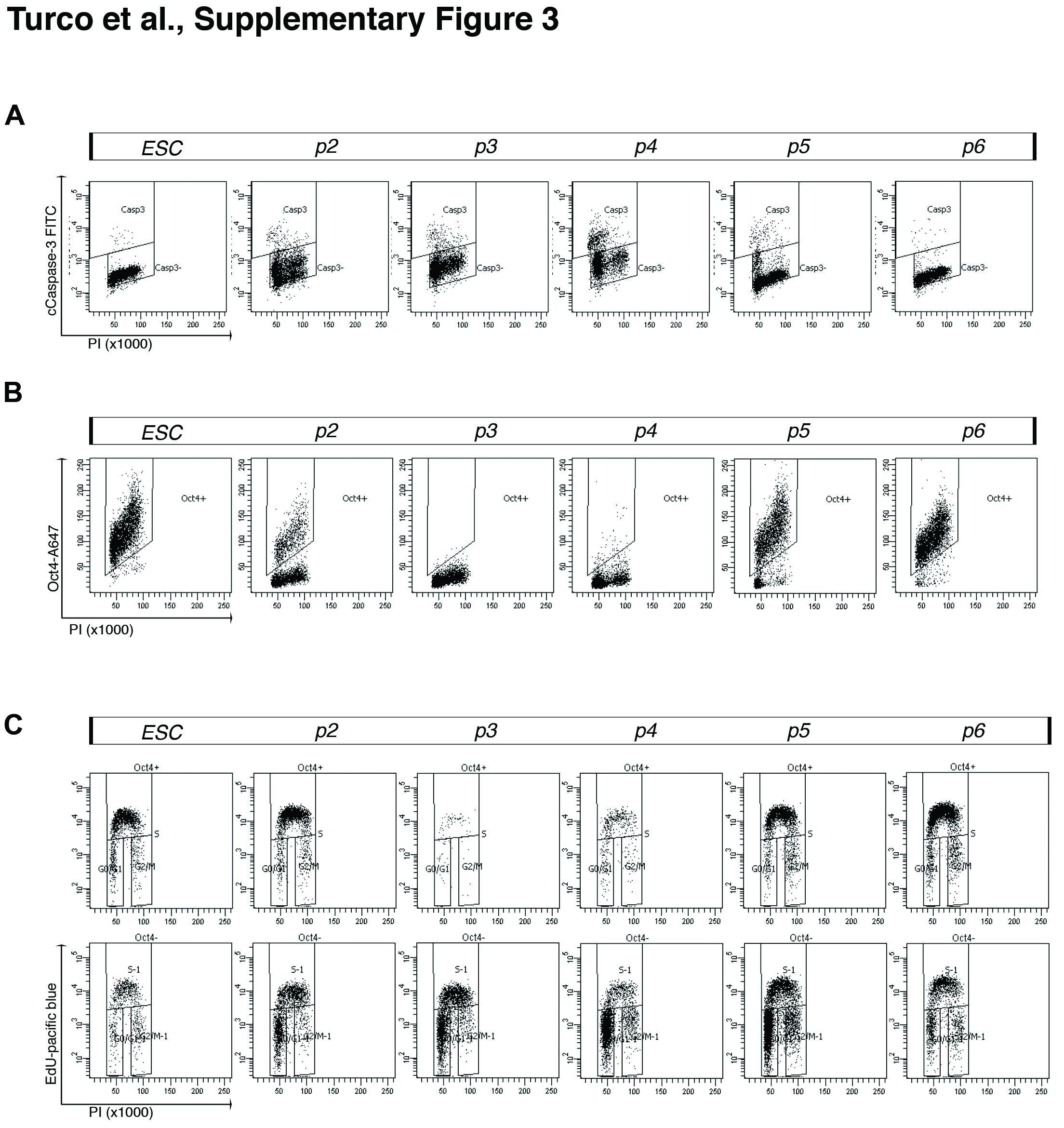

Supplement: Supplementary file 3 [file stem0030-2423-SD3.tif]

# Turco et al., Supplementary Figure 5

**A**

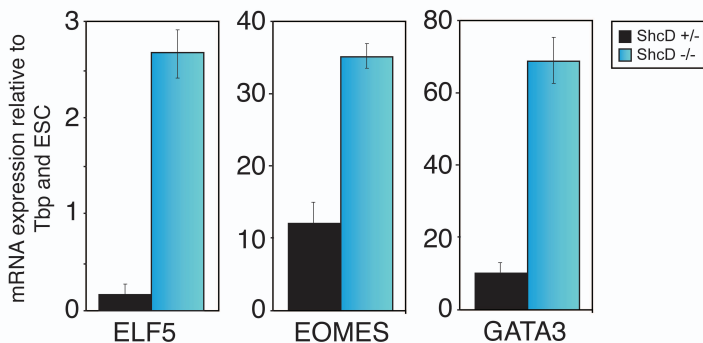

**B**

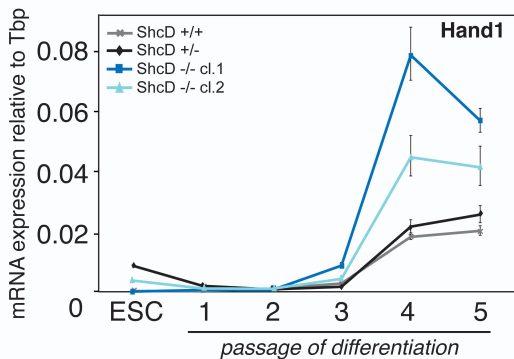

Supplement: Supplementary file 5 [file stem0030-2423-SD5.pdf]

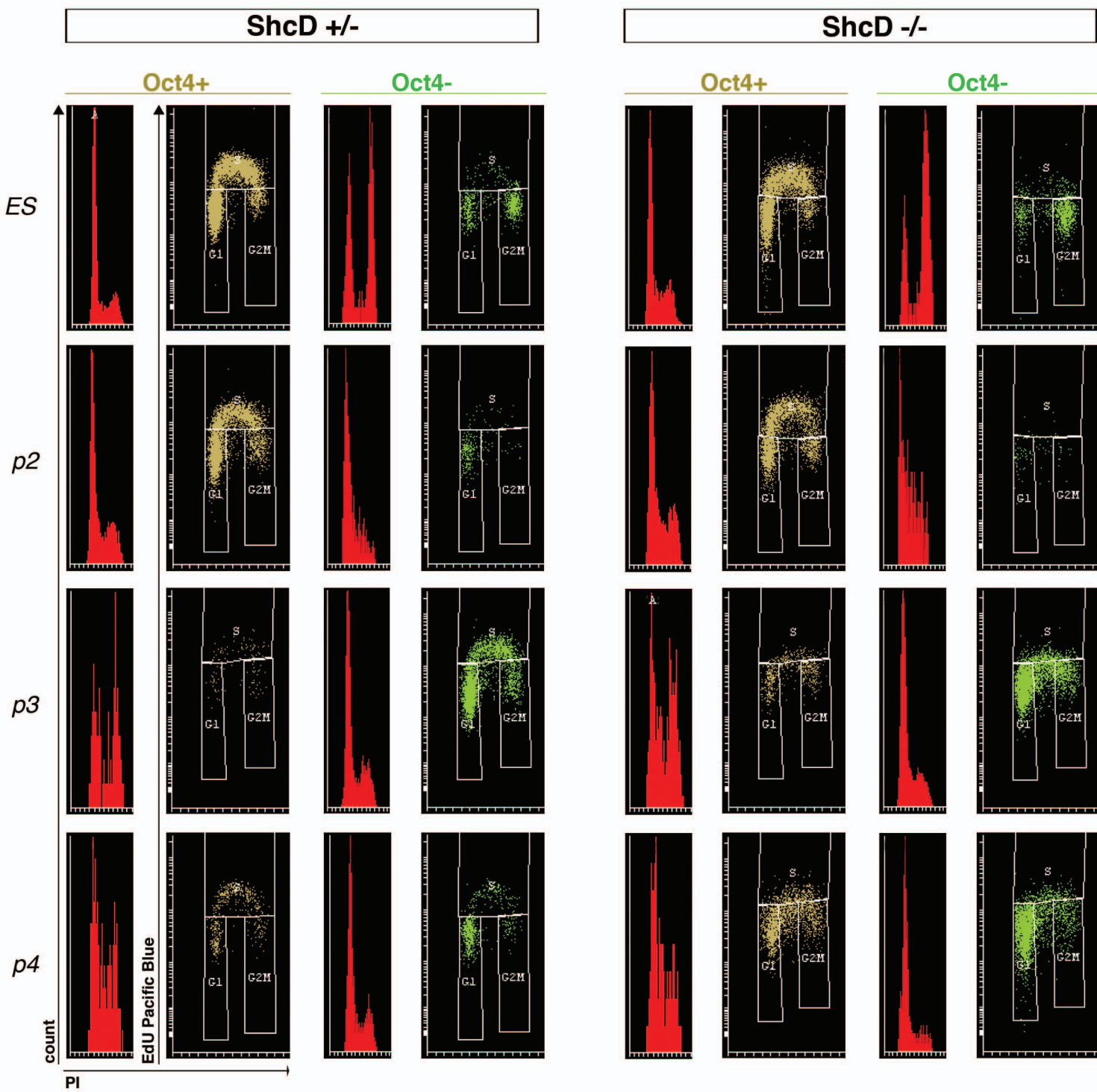

Supplement: Supplementary file 6 [file stem0030-2423-SD6.pdf]

**A**

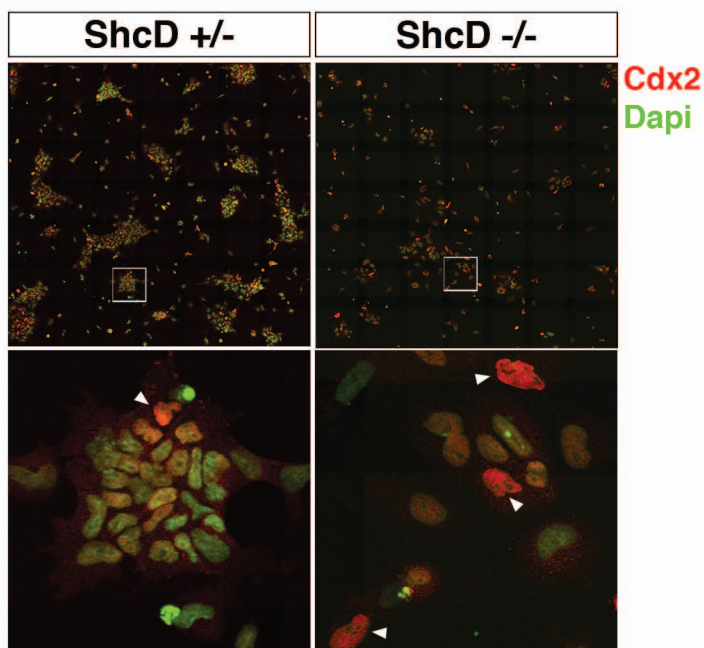

**B**

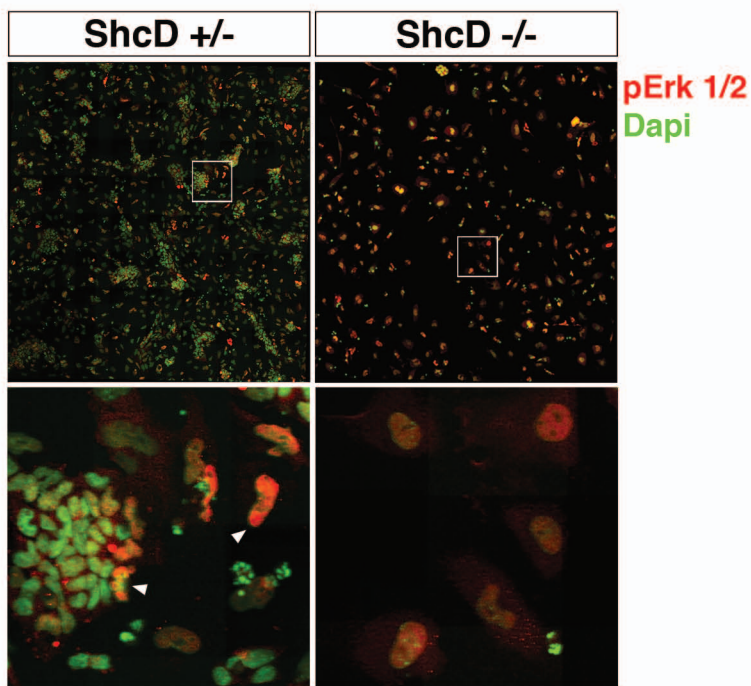

Supplement: Supplementary file 7 [file stem0030-2423-SD7.pdf]

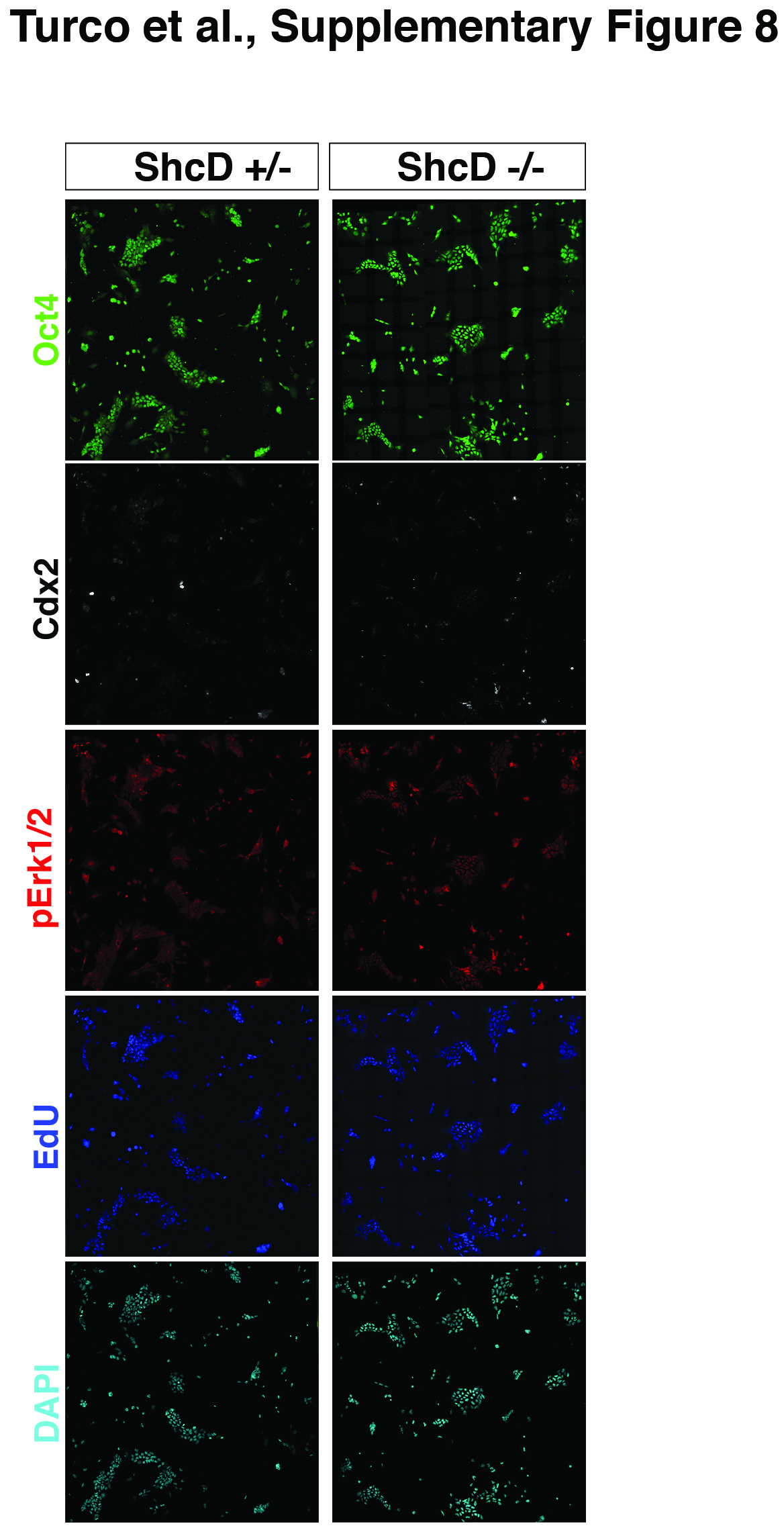

Supplement: Supplementary file 8 [file stem0030-2423-SD8.tif]
