# Supplementary material for: Cellular Heterogeneity During Embryonic Stem Cell Differentiation to Epiblast Stem Cells is Revealed by the ShcD/RaLP Adaptor Protein
Source: Stem Cells. 2012 Sep 4;30(11):2423–36. doi: 10.1002/stem.1217 (PMC3533801; doi:10.1002/stem.1217)
Supplement: Supplementary file 13 [file stem0030-2423-SD13.pdf]

Cellular Heterogeneity during Embryonic Stem Cell Differentiation to Epiblast Stem Cells is Revealed by the ShcD/RaLP Adaptor Protein

Margherita Y. Turco, Laura Furia, Anja Dietze, Luis Fernandez Diaz, Simona Ronzoni, Anna Sciullo, Antonio Simeone, Daniel Constam, Mario Faretta, Luisa Lanfrancone

SUPPORTING INFORMATION

This supporting information contains a part of the materials and methods, 8 supplementary figures and 4 time-lapse movies.

MATERIALS AND METHODS

Time-lapse Microscopy and Analysis

For time-lapse imaging, Oct4-GFP ESCs were plated in ESC medium onto gelatinized gridded glass bottom coverslip dishes (MatTek Corporation) and were placed in a humidified culture chamber at 37°C and 5% CO_2. Cells were imaged by microscopy Nikon Eclipse Ti PFS microscope system (Nikon) equipped with a Roper Cascade 1K CCD camera (Photometrics) and controlled by Metamorph software (Molecular Devices). Partially overlapping fields were collected with a 20X 0.75 NA objective every 30 mins for 24 h in both fluorescence (GFP) and transmitted light (phase contrast) channels. Immediately after live-cell imaging, cells were fixed and immunostained for Cdx2 and Oct4 detection. The stained coverslip was then acquired employing the Leica SP5 confocal system using the tiling procedure with the confocal control software. Laser reflection signal allowed visualization of the photoprinted

grid. Cells were thus relocated based on the visual comparison of the acquired area comparing the mosaic from the fixed cells and the last time-lapse prefixation timepoint. Image analysis was performed using ImageJ software (W. Rasband, National Institute of Health).

Real-Time Reverse Transcription-Polymerase Chain Reaction

Total RNA was extracted using the RNeasy Mini kit with on column DNAse treatment (Qiagen), following manufacturer's instructions. RNA quality and concentration were determined using the Nanodrop ND-1000 Spectrophotometer (Thermo Scientific). 1 μg of total RNA was reverse transcribed using Superscript III Reverse Transcriptase (Invitrogen) according to manufacturer's instructions. Quantitative real-time PCR (qRT-PCR) was performed on 7900HT Fast Real-Time PCR system (Applied Biosystems) using Fast SYBR Green PCR Master Mix or TaqMan Gene expression assays (Applied Biosystems). Primer sequences for SYBR green reactions are available upon request.

Western blotting

The ShcD antibody was generated in collaboration with the Biochemistry Unit at the IFOM-IEO campus. Antibodies against the CH2 domain of murine ShcD was raised using the peptide sequence of 16 amino acids: Nter-QPYRKYDNTGLLPPKK-Cter [8]. Cell lysates were lysed in ice cold Radio Immuno Precipitation Assay (RIPA) buffer (150 mM sodium chloride, 1% Triton X-100, 0.5% sodium deoxycholate, 0.1% SDS and 50 mM Tris pH8.0) supplemented with protease inhibitor cocktail Complete Mini (Roche), phosphatase inhibitor mix PhosSTOP (Roche) and 50 mM Dithiothreitol (DTT). Protein was resolved by SDS-polyacrylamide gel

electrophoresis and transferred onto a poly-vinylidene fluoride (PVDF) Immobilon™-P transfer (Millipore) membranes. Membranes were hybridized with anti-phospho-Erk1/2 Thr202/Tyr204 (Cell signalling; #9106), anti-phospho-SMAD2 Ser465/467 (Cell Signaling; #3108) antibodies and were detected using horseradish peroxidase conjugated IgG as secondary antibody and Amersham ECL Plus Western Blotting Detection Reagents (GE Healthcare) following manufacturer's instructions. Membranes were stripped in Stripping Buffer (2% SDS, 62.5 mM Tris pH6.8 in water) freshly added with 100 mM β-mercaptoethanol (BDH) and reprobed for Erk1/2 (Santa Cruz; sc94) or SMAD2 (Cell Signaling; #3103).

***In Situ* Hybridization Assays**

Whole-mount *in situ* hybridization of embryos from E4.5 to E7.5 and E10.5 brain was performed using digoxigenin-labelled RNA probes (Boehringer), as described [9, 10]. For embryos at E.10.5, E12.5 and E16.5, *in situ* hybridization experiments were performed using radiolabelled riboprobes, as described [11]. The ShcD antisense probe was synthesized using the ShcD cDNA template comprising exons 1 to 4 (611bp, NM_ 199022.2).

REFERENCES

1. Burdon T, Chambers I, Stracey C, et al. Signaling mechanisms regulating self-renewal and differentiation of pluripotent embryonic stem cells. Cells Tissues Organs. 1999;165:131-143.
2. Buehr M, Smith A. Genesis of embryonic stem cells. Philos Trans R Soc Lond B Biol Sci. 2003;358:1397-1402; discussion 1402.
3. Ying QL, Stavridis M, Griffiths D, et al. Conversion of embryonic stem cells into neuroectodermal precursors in adherent monoculture. Nat Biotechnol. 2003;21:183-186.
4. Ying QL, Wray J, Nichols J, et al. The ground state of embryonic stem cell self-renewal. Nature. 2008;453:519-523.
5. Ying QL, Smith AG. Defined conditions for neural commitment and differentiation. Methods Enzymol. 2003;365:327-341.
6. Conti L, Pollard SM, Gorba T, et al. Niche-independent symmetrical self-renewal of a mammalian tissue stem cell. PLoS Biol. 2005;3:e283.
7. Guo G, Yang J, Nichols J, et al. Klf4 reverts developmentally programmed restriction of ground state pluripotency. Development. 2009;136:1063-1069.
8. Jones N, Hardy WR, Friese MB, et al. Analysis of a Shc family adaptor protein, ShcD/Shc4, that associates with muscle-specific kinase. Mol Cell Biol. 2007;27:4759-4773.
9. Varlet I, Collignon J, Robertson EJ. nodal expression in the primitive endoderm is required for specification of the anterior axis during mouse gastrulation. Development. 1997;124:1033-1044.
10. Beck S, Le Good JA, Guzman M, et al. Extraembryonic proteases regulate Nodal signalling during gastrulation. Nat Cell Biol. 2002;4:981-985.
11. Simeone A. Detection of mRNA in tissue sections with radiolabelled riboprobes. In: Wiles MV, ed. In Situ Hybridization, A Practical Approach, 2nd ed. . Oxford: Oxford University Press; 1999:69-86.
